# Policies to prevent zoonotic spillover: a systematic scoping review of evaluative evidence

**DOI:** 10.1186/s12992-023-00986-x

**Published:** 2023-11-08

**Authors:** Chloe Clifford Astbury, Kirsten M. Lee, Ryan Mcleod, Raphael Aguiar, Asma Atique, Marilen Balolong, Janielle Clarke, Anastassia Demeshko, Ronald Labonté, Arne Ruckert, Priyanka Sibal, Kathleen Chelsea Togño, A. M. Viens, Mary Wiktorowicz, Marc K. Yambayamba, Amy Yau, Tarra L. Penney

**Affiliations:** 1 School of Global Health, York University, Toronto, ON Canada; 2 Dahdaleh Institute for Global Health Research, York University, Toronto, ON Canada; 3 Global Strategy Lab, York University, Toronto, ON Canada; 4 Applied Microbiology for Health and Environment Research Group, College of Arts and Sciences, University of the Philippines Manila, Manila, Philippines; 5 School of Epidemiology and Public Health, University of Ottawa, Ottawa, ON Canada; 6 School of Health Policy and Management, York University, Toronto, ON Canada; 7 School of Public Health, University of Kinshasa, Kinshasa, Democratic Republic of Congo; 8 Department of Public Health, Environments and Society, London School of Hygiene & Tropical Medicine, London, UK

**Keywords:** Zoonotic spillover, One health, Public policy, Evaluation, Emerging zoonoses, Deep prevention

## Abstract

**Background:**

Emerging infectious diseases of zoonotic origin present a critical threat to global population health. As accelerating globalisation makes epidemics and pandemics more difficult to contain, there is a need for effective preventive interventions that reduce the risk of zoonotic spillover events. Public policies can play a key role in preventing spillover events. The aim of this review is to identify and describe evaluations of public policies that target the determinants of zoonotic spillover. Our approach is informed by a One Health perspective, acknowledging the inter-connectedness of human, animal and environmental health.

**Methods:**

In this systematic scoping review, we searched Medline, SCOPUS, Web of Science and Global Health in May 2021 using search terms combining animal health and the animal-human interface, public policy, prevention and zoonoses. We screened titles and abstracts, extracted data and reported our process in line with PRISMA-ScR guidelines. We also searched relevant organisations’ websites for evaluations published in the grey literature. All evaluations of public policies aiming to prevent zoonotic spillover events were eligible for inclusion. We summarised key data from each study, mapping policies along the spillover pathway.

**Results:**

Our review found 95 publications evaluating 111 policies. We identified 27 unique policy options including habitat protection; trade regulations; border control and quarantine procedures; farm and market biosecurity measures; public information campaigns; and vaccination programmes, as well as multi-component programmes. These were implemented by many sectors, highlighting the cross-sectoral nature of zoonotic spillover prevention. Reports emphasised the importance of surveillance data in both guiding prevention efforts and enabling policy evaluation, as well as the importance of industry and private sector actors in implementing many of these policies. Thoughtful engagement with stakeholders ranging from subsistence hunters and farmers to industrial animal agriculture operations is key for policy success in this area.

**Conclusion:**

This review outlines the state of the evaluative evidence around policies to prevent zoonotic spillover in order to guide policy decision-making and focus research efforts. Since we found that most of the existing policy evaluations target ‘downstream’ determinants, additional research could focus on evaluating policies targeting ‘upstream’ determinants of zoonotic spillover, such as land use change, and policies impacting infection intensity and pathogen shedding in animal populations, such as those targeting animal welfare.

**Supplementary Information:**

The online version contains supplementary material available at 10.1186/s12992-023-00986-x.

## Background

The increasing incidence of zoonotic emerging infectious diseases (EIDs) has been attributed to behavioural practices and ecological and socioeconomic change, and is predicted to continue in the coming years [1]. Higher levels of anthropogenic activity, including agricultural intensification, urbanisation and other forms of land use change, have led to increased interactions between wildlife, humans and livestock, increasing the risk of cross-species transmission [2–4]. Meanwhile, accelerating rates of globalisation and urbanisation, leading to increased global movement of people and goods and more dense human settlements, have made outbreaks of disease in human populations more difficult to contain [5]. In response, a call has been issued by leading organisations and experts, including the United Nations Environment Programme, the International Livestock Research Institute and the Intergovernmental Science-Policy Platform on Biodiversity and Ecosystem Services, to complement reactive policy responses with policies that prevent zoonotic EIDs [1, 6–10]. This approach, sometimes called deep prevention, would need to target upstream drivers to reduce the risk of outbreaks occuring [11].

**Fig. 1:**
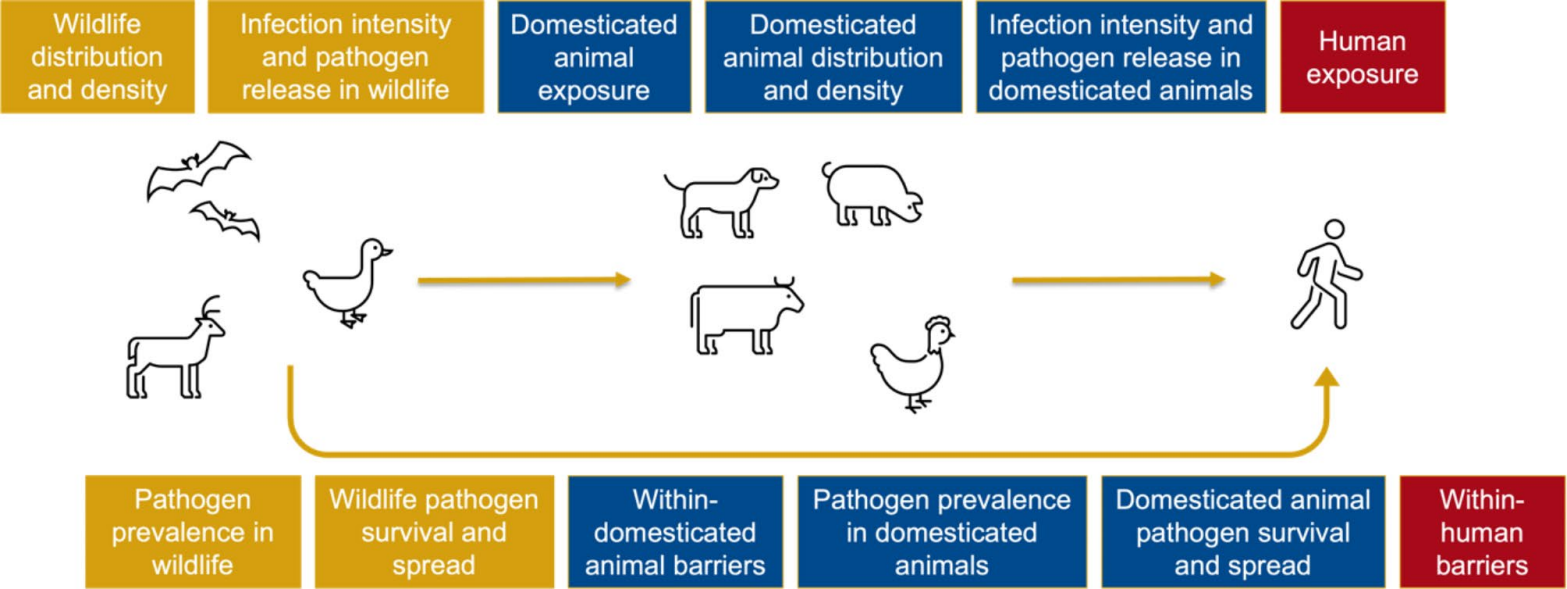
Spillover pathway adapted from Plowright et al. [12, 13]

Zoonotic spillover, defined as the transmission of a pathogen from an animal to a human, depends on the alignment of ecological, epidemiological and behavioural factors [12]. Zoonotic pathogens must be transmitted across a spillover pathway (Fig. 1) in order to induce infections in humans [12, 13]. This involves meeting a series of conditions including appropriate density and distribution of reservoir hosts, pathogen prevalence, infection intensity and human exposure [12]. Across this pathway, a number of drivers of zoonotic spillover have been identified, including changes in wildlife and livestock populations [14]; deforestation, urbanisation and other forms of land use change [15, 16]; bushmeat consumption [17–19]; and a variety of human practices including hunting, farming, animal husbandry, mining, keeping of exotic pets and trade [8, 9, 20–22]. These large-scale changes have repeatedly given rise to spillover events [2, 15, 23], sometimes involving pathogens with epidemic or pandemic potential [24].

The responsibility for addressing zoonotic disease frequently spans multiple sectors of governance due to its relevance for both animals and humans. A One Health perspective, which recognises the health of humans, animals and the environment as being closely linked and inter-dependent [25], can be useful in understanding the spillover pathway and drivers of spillover events, as well as informing policy and governance approaches to address this cross-sectoral problem. At the international level, the World Health Organization, the Food and Agriculture Organization, the World Organisation for Animal Health and the United Nations Environment Programme have endorsed a One Health approach to policymaking to respond to zoonotic infectious diseases, emphasising collaboration between agencies [26].

### Operationalising a One Health approach to policy

While One Health is a promising approach to preventing zoonotic EIDs, operationalising this concept remains a challenge. Evaluative evidence exists around the effectiveness of interventions to prevent spillover events [13, 27–29], however these have often been implemented as short- to medium-term programmes or academic investigations [8]. In some cases, zoonoses have re-emerged after successful programmes have ended [29]. As a result, experts have argued for the incorporation of successful interventions into policy frameworks, providing interventions with the sustainability required for long-term disease control [8, 10].

Operationalising a One Health approach to policy involves understanding the policy options, identifying the stakeholders involved and developing insights into how to successfully implement and evaluate these policies. Although the longevity and scope of government actions may make policy an effective vehicle for prevention of emerging diseases, implementing policy is a complex process involving numerous actors with competing views and interests [30]. This context presents challenges for policy development and implementation. Where relevant policies are designed and implemented in isolation, opportunities for co-benefits may be missed and interventions may produce unintended consequences [31]. Finally, while evaluative evidence is key to informing future policy decisions, the complex systems in which policies are often implemented make evaluation challenging [32].

### Aims and scope

To provide insights around how to use policy to successfully prevent zoonotic spillover events, it is necessary to synthesise the available evaluative evidence. A One Health perspective allows this evidence synthesis to incorporate a wide range of policy instruments and actors and to identify approaches to successfully implementing and evaluating policies in this complex, multi-sectoral context.

Approaches to managing epidemic and pandemic infectious pathogens when they have entered human populations have been systematically catalogued in the medical literature [33–39]. These measures include hand washing, face masks, school closures, contact tracing, vaccination and case isolation. Further upstream, systematic reviews of interventions targeting the spillover pathway have predominantly focused on programmes rather than policies, and have been restricted by various characteristics such as geographic region [28] or pathogen type [29], or focused on programmes with an explicit endorsement of a One Health approach [27]. In consequence, a comprehensive understanding of what policies to prevent zoonotic spillover have been evaluated, what actors are involved, and how to successfully implement and evaluate them, is lacking. To address these research gaps, our objective was to synthesise the existing evaluative evidence around policies that target the determinants of zoonotic spillover.

Our approach to identifying and analysing this literature was informed by a One Health perspective, acknowledging the inter-connectedness of human, animal and environmental health.

## Methods

We conducted a systematic scoping review of evaluations of policies aimed at preventing zoonotic spillover events, based on a previously published protocol [40]. Results are reported in accordance with the Preferred Reporting Items for Systematic Reviews and Meta-Analyses Extension for Scoping Reviews [41]. The scoping review was conducted in line with guidelines published by Arksey and O’Malley and refined by Levac and colleagues [42–44], which emphasise an iterative approach suited to an exploratory research question.

The One Health perspective guided the development of the review methodology. This included the search strategy and inclusion criteria, which allow for the inclusion of policies focused on human, animal or environmental health (or any combination of these areas) and with leadership from one or more of these sectors, and the research questions, which seek to outline the policies and the range of sectors involved in implementation. While our focus on the spillover pathway meant we only included policies that had been evaluated in terms of their impacts on animal and human population distributions, health and interactions, we explicitly searched for environment-focused policies (e.g., protection of wetlands and other wildlife habitats) that might have been evaluated from this perspective. We also aimed to interrogate the One Health approach to governance, by assessing to what extent cross-sectoral collaboration – a key tenet of One Health practice [25] – emerged as a reason for policy success.

### Stage 1: identifying the research question

Informed by our research objective, our research questions were:

- What policies aimed at preventing zoonotic spillover (i.e., policies that target the determinants of zoonotic spillover included in the spillover pathway [12]: population distribution, health and interactions) have been evaluated?
  - What are the types of policies?
  - Which policy actors (single department, multi-sectoral, whole of government) are involved?
- What are the reasons for policy success and failure, and the unintended consequences of implementing these policies?
- How has evaluation of these policies been approached in the literature?
  - What are the methods or study designs used?
  - What are the outcomes?
  - What are the opportunities and challenges for evaluation?

### Stage 2: identifying relevant studies

We systematically searched four electronic databases (Medline, Scopus, Web of Science, Global Health) in May 2021. The search strategy was organized by the main concepts in our research question: the spillover pathway; public policy; prevention; and zoonotic pathogens. The search strategy was developed iteratively, informed by existing systematic reviews focused on related concepts [28, 45–49] and known indicator papers meeting inclusion criteria. We also searched the websites of 18 organisations involved in the prevention of zoonotic spillover to identify relevant grey literature. The choice of organisations was informed by an actor mapping exercise in which we identified key international organisations working on the prevention of emerging zoonoses using network sampling [50]. We searched the websites of a subset of these organisations, focusing on inter-governmental organisations and organisations whose main focus was zoonotic disease. See Supplementary File 1 for details of academic database and grey literature search strategies.

### Stage 3: study selection

Studies were included if they met the following criteria:

- Primary empirical study with an English-language abstract from any country or region (reviews were excluded);
- Study reporting empirical findings from an evaluation of any sort; and.
- Study focused on a policy implemented by government that targets the determinants of zoonotic spillover.

Academic records identified through the searches were collated and double screened using the online platform Covidence [51]. Two researchers (CCA and KML) initially screened titles and abstracts. Title and abstract screening of an initial set of 100 papers was undertaken by both researchers independently. Results were compared to ensure consistency in decisions around study eligibility, and discrepancies were resolved through consensus. This process was repeated until an acceptable level of agreement (> 90%) was reached. The remaining papers were then screened by one of the two reviewers. Full-text screening was undertaken by two independent researchers and discrepancies were resolved by consensus. Studies with full-texts in any language were eligible for inclusion if they include an English-language abstract. Full-text studies published in French, Spanish or Chinese were single-screened by a member of the research team fluent in that language (CCA or AY). Studies published in other languages were translated as necessary.

Grey literature was screened by one researcher (CCA) to determine whether it met the inclusion criteria. Publications were initially screened by looking at titles, tables of contents and executive summaries. Where these indicated that the publication might be eligible, documents were read in full to determine if inclusion criteria were met.

In line with published guidelines, the approach to study selection was refined iteratively when reviewing articles for inclusion [42–44].

### Stage 4: charting the data

Data charting was conducted using a form designed to identify the information required to answer the research question and sub-research questions (see Supplementary File 2). Data charting focused on characteristics of the study, the policy, and the evaluation. For each policy, this included identifying which determinant of zoonotic spillover situated along the spillover pathway was being targeted. For the purpose of this study, we used a model of the spillover pathway adapted from Plowright et al.’s work [12, 13], in which we differentiated between wildlife and domesticated animals (Fig. 1). This differentiation is important in the policy context, as the wildlife-domesticated animal interface is an important site for intervention, as well as the human-animal interface.

The data charting form was piloted with ten records to ensure that it was consistent with the research question, and revised iteratively [42–44]. Data charting was conducted by one researcher (CCA, RM, JC, AD or PS) and checked by a second researcher (CCA or KML). Discrepancies were resolved by consensus.

### Stage 5: collating, summarising and reporting the results

Our protocol stated that we would use the Quality Assessment Tool for Quantitative Studies developed by the Effective Public Health Practice Project [52] to assess study quality [40]. However, on reviewing the included studies we selected two tools that were more appropriate to their characteristics: (1) ROBINS-I [53] for quantitative outcome evaluations and (2) a tool developed by the authors of a previous review [54] – based on Dixon-Woods et al.’s approach to assessing study credibility and contribution [55] – for all other study types. Two researchers (CCA and KML) assessed study quality independently for an initial set of 10 studies, before comparing assessments and reaching agreement where discrepancies occurred. This process was repeated until an adequate level of agreement was reached (> 90%). The remaining studies were assessed by a single researcher (CCA or KML). Records were not excluded based on quality assessment. Instead, assessments were primarily used to help synthesize the literature on how policies were evaluated. Quality assessment was not performed on grey literature due to the wide variability in the format and comprehensiveness of included publications.

We analysed the charted data, presenting a numerical summary of the included studies in table form, allowing us to describe the range of policy interventions that have been evaluated, aspects of policy implementation and approaches to evaluation. Based on the charted data, we inductively grouped evaluated policies with similar characteristics into policy types and assigned a policy instrument to each policy type: communication/marketing, guidelines, fiscal, regulation, legislation, environmental/social planning or service provision. We mapped policy types onto the spillover pathway shown in Fig. 1 to outline the policies that have been used to target each of these determinants. Thematic analysis was conducted using the approach described by Braun and Clarke where the focus is guided by the researcher’s analytic interests [56], with five overarching themes chosen as an a priori coding framework: (1) reasons for policy success; (2) reasons for policy failure; (3) unintended consequences of policy implementation; (4) opportunities for policy evaluation; and (5) challenges for policy evaluation. We selected these themes based on our research questions and previous familiarisation with the included articles during the process of article selection, data extraction and quality assessment. Sub-themes were subsequently identified through close reading and coding of the included articles. Thematic analysis was conducted by one researcher (RM) using the qualitative data analysis software Dedoose [57] and reviewed by the lead author (CCA).

## Results

### Study characteristics

**Fig. 2:**
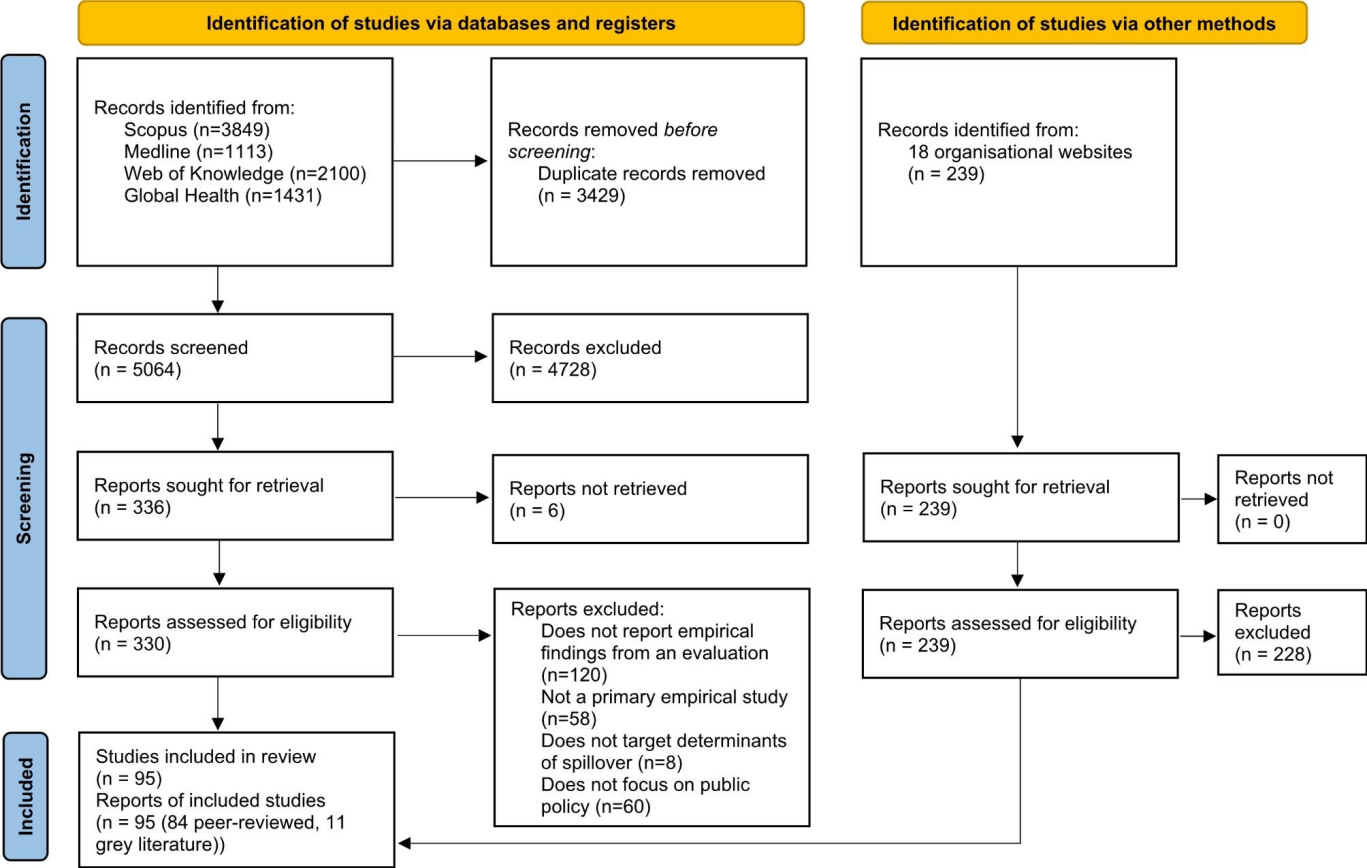
PRISMA 2020 diagram [58]

After removing duplicates, our searches identified a total of 5064 academic records. After screening titles and abstracts, we considered 330 records for full-text review. We also identified 11 relevant publications through our grey literature search. Grey literature reports were published by five organisations: four organisations focused on health and disease, including an intergovernmental organisation (the World Organisation for Animal Health) and three non-governmental organisations (the One Health Commission, the Global Alliance for Rabies Control and EcoHealth Alliance); and one non-governmental organisation focused on wildlife trade (TRAFFIC). In total, we included 95 publications in this review (PRISMA diagram in Fig. 2) [58].

We excluded studies which assessed the unintended consequences of policies to prevent zoonotic spillover without evaluating their effectiveness. This included studies that looked exclusively at the mental health impacts of mandatory livestock culls on farm workers [59]; studies which focused on potentially relevant factors, such as the wildlife trade, but with no consideration of outcomes situated on the spillover pathway [60]; and studies which assessed the detection power of surveillance systems without assessing the impact of associated policy interventions [61–63].

### Policy characteristics

The characteristics of the policies evaluated in the included studies are presented in Supplementary File 3 and summarised in Table 1. Some studies evaluated more than one policy, particularly modelling studies which compared the impacts of several policy options and process evaluations focused on a range of activities undertaken by a single government. Therefore, the number of evaluated policies (n = 111) is greater than the number of included studies (n = 95).

Most policies were evaluated for their impact on human exposure (21%), pathogen prevalence in domesticated animals (18%), barriers within domesticated animals (15%), and pathogen survival and spread in domesticated animals (9%). There were also a number of multi-component policies studies across multiple stages of the spillover pathway (18%). Fewer studies focused on wildlife health and populations, and none of the included studies evaluated policies for their impact on infection intensity and pathogen release in either domesticated animals or wildlife.

Where the government department responsible for implementing a policy was identified in the paper, most policies were implemented by a single department (35%), although there were a number of multi-sectoral efforts (24%). The range of government sectors responsible for implementing policies to prevent zoonotic spillover included human health, animal health, food safety, agriculture, conservation, national parks, forestry, fisheries, environmental protection, border control and foreign affairs. Policies were predominantly intended to be implemented by private sector actors, including individuals and organisations working in trade, retail, hunting and animal agriculture. However, some policies were also implemented by public sector actors working in public health, veterinary public health and environmental conservation.

Most policies were situated in high-income (49%) and upper middle-income (28%) countries, with studies from East Asia and the Pacific (43%) and Europe and Central Asia (19%) dominating. Publications focused on policies targeting various zoonotic diseases, with the most common being avian influenza (50%), rabies (19%), brucellosis (11%) and Hendra virus (4%).

Most policies were evaluated using process (38%) or outcome (31%) evaluation. The most frequently used policy instrument was legislation (59%), particularly for managing pathogen spread in domesticated animals through measures such as mandatory vaccination, culls or disinfection protocols. Meanwhile, communication and marketing or service provision was more typically used to reduce risk in wildlife and human populations, for example by providing guidance around recommended hygiene protocol, by distributing oral vaccination in wildlife habitat or by offering vaccination to human populations.

**Table 1:** Characteristics of evaluated policies (n) across the spillover pathway^1^

Characteristics	Total (n (%))			Spillover pathway stage									
		Wildlife distribution and density	Pathogen prevalence		Pathogen survival and spread	Domesticate animal exposure	Within-domesticated animal barriers	Domesticated animal distribution and density	Pathogen prevalence	Pathogen survival and spread	Human exposure	Within-human barriers	Multiple stages
**Target population**		**Wildlife**				**Domesticated animals**					**Humans**		**Multiple**
Total (n (%))	111 (100)	5 (5)	7 (6)		1(1)	4 (4)	17 (15)	1 (1)	18 (16)	10 (9)	23 (21)	5 (5)	20 (18)
**Policy and governance level**													
Local	34 (31)	1	3		0	2	4	1	0	4	15	0	4
National	54 (49)	2	3		1	4	8	0	14	3	5	5	11
Regional (e.g., European region)	9 (8)	0	0		0	0	2	0	3	1	1	0	2
Global	6 (7)	1	0		0	0	1	0	0	1	0	0	3
**Policy and governance sector**													
Animal health	18 (16)	0	0		0	3	3	0	6	1	1	0	4
Environment	5 (5)	1	1		1	0	0	0	1	1	0	0	0
Human health	17 (15)	0	0		0	0	2	0	0	1	9	4	1
Multi-sectoral	26 (23)	2	2		0	0	3	0	4	2	6	1	6
Other or not stated	45 (41)	2	4		0	1	9	1	7	5	7	0	9
**Implementing sector**													
Retail	19 (17)	0	0		0	0	0	1	0	5	13	0	0
Trade	7 (6)	0	1		0	0	0	0	2	1	3	0	0
Farming	47 (42)	2	0		1	4	13	0	10	4	3	0	10
Human health	8 (7)	0	0		0	0	1	0	0	0	0	5	2
Animal health	8 (7)	0	0		0	0	3	0	2	0	1	0	2
Conservation	9 (8)	3	6		0	0	0	0	0	0	0	0	0
Other or multiple	13 (12)	0	0		0	0	0	0	4	0	3	0	6
**Country income**													
High	52 (47)	2	7		0	3	5	0	11	5	7	3	9
Upper middle	31 (28)	2	0		1	1	4	1	3	4	14	0	1
Lower middle	23 (21)	1	0		0	0	7	0	4	1	1	2	7
Low	2 (2)	0	0		0	0	0	0	0	0	1	0	1
**Region**													
East Asia and Pacific	46 (41)	1	1		0	2	4	1	8	7	18	2	2
Europe and Central Asia	21 (19)	1	1		0	2	4	0	4	2	2	1	4
Latin America & the Caribbean	4 (4)	1	0		0	0	3	0	0	0	0	0	0
Middle East and North Africa	4 (4)	0	0		0	0	2	0	1	0	0	0	1
North America	17 (15)	1	5		0	0	1	0	1	1	2	2	4
South Asia	2 (2)	1	0		0	0	0	0	0	0	0	0	1
Sub-Saharan Africa	14 (13)	0	0		1	0	2	0	4	0	1	0	6
Global	1 (1)	0	0		0	0	1	0	0	0	0	0	0
**Disease**													
Avian influenza	55 (50)	1	0		0	2	5	1	6	9	18	12	12
Brucellosis	12 (11)	1	0		0	0	3	0	4	0	3	1	1
Hendra virus	4 (4)	0	0		0	2	1	0	0	0	1	0	0
Rabies	21 (19)	2	6		0	0	6	0	2	0	0	2	2
Other	19 (17)	1	1		1	0	2	0	6	1	1	5	5
**Evaluation type**													
Formative	18 (16)	1	3		0	0	3	0	6	0	0	0	5
Process	42 (38)	0	0		0	4	4	0	9	4	9	3	9
Outcome	35 (32)	2	2		1	0	5	1	3	6	14	0	1
Economic	13 (12)	1	2		0	0	3	0	0	0	0	2	5
Impact	3 (3)	1	0		0	0	2	0	0	0	0	0	0
**Policy instrument**													
Communication/marketing	6 (5)	0	0		0	1	0	0	0	1	4	0	0
Regulation	4 (4)	0	0		1	0	1	0	0	0	2	0	0
Legislation	65 (59)	3	1		0	2	14	1	16	8	16	0	0
Service provision	16 (14)	2	6		0	0	1	0	1	0	0	5	1
Multiple instruments	20 (18)	0	0		0	1	1	0	1	1	1	0	15

^1^Infection intensity and pathogen release in both wildlife and domesticated animals are excluded from the table as no relevant studies were identified; policy numbers do not add up to 111 for all characteristics (country income, region and governance level), reflecting modelling studies where this contextual information was not provided as framing for the study

### What policies aimed at preventing zoonotic spillover have been evaluated?

Policy types targeted different determinants across the pathway to zoonotic spillover and used various approaches with different evidence of success (Table 2). We identified policy options including culling – both general and targeted – of wild and domesticated animals; habitat protection (limiting activities such as agriculture and animal husbandry in wildlife habitats); supplemental feeding to control wildlife movements; vaccination of both wildlife, domesticated animals and human populations with occupational exposure to animals; policies to improve biosecurity in sites where animals are kept, slaughtered and sold, including mandates and information campaigns; live animal market closures; and bans on hunting and selling wildlife. Where outcomes or impacts were evaluated, most policies saw some level of success (i.e., outcome measures were found to vary in a direction that indicated policy success), though relative effectiveness was not assessed due to variation in study design and outcome measure. Policies with consistent evidence of effectiveness – where outcome measures varied in a direction that indicated policy success in all studies included in the review – included culling and sterilisation of wildlife populations, habitat protection, vaccination in wildlife and domesticated animal populations and mandated disinfection protocols. Policies with equivocal evidence of success (i.e., outcome measures varied in different directions or studies had different findings, some indicating success and some indicating failure) included supplemental feeding of wildlife, pre-emptive livestock culls, live animal market closures and bans on wildlife hunting, trade and consumption. For many policies, there were no impact or outcome evaluations identified in this review.

**Table 2:** Policies identified across the pathway to zoonotic spillover, adapted from [12, 13]

Stage in pathway	Policy types and example studies	Policy instrument	Policy success^1^
Wildlife distribution and density	Culling of wildlife populations [64]	Service provision	Success
	Sterilisation of wildlife populations [64]	Service provision	Success
	Supplemental feeding of wildlife [65]	Service provision	Equivocal
	Habitat protection [66]	Legislation	Success
Pathogen prevalence in wildlife	Vaccination campaign using oral bait [67–69]	Service provision	Success
	Border surveillance and biosecurity [70]	Legislation	Success
Infection intensity and pathogen release in wildlife	None identified	N/A	N/A
Wildlife pathogen survival and spread	Regulations around disposing of infected wildlife carcasses [71]	Regulation	Success
Domesticated animal exposure	Mandated separation of wildlife and livestock [72]	Legislation	N/A
	Ban on feeding catering waste to livestock [73]	Legislation	N/A
	Information leaflets to change animal owner behaviour (e.g., stabling animals overnight, placing feed and water away from wooded areas where wildlife live) [74, 75]	Communication/marketing	N/A
Within-domesticated animal barriers	Vaccination of livestock or other domesticated animals [74, 76–78]	Service provision (providing government veterinarians offering free vaccination) Legislation (mandating livestock vaccinations to be undertaken by owners)	Success
Domesticated animal distribution and density	Limits on live animal market size [79]	Legislation	Success
Pathogen prevalence in domesticated animals	Animal quarantine (testing, prophylaxis, culling of infected animals) [80]	Legislation	N/A
	Screen and cull of infected animals [81–83]	Legislation	Success
	Pre-emptive cull (e.g. ring cull, general cull) [84, 85]	Legislation	Equivocal
Infection intensity and pathogen release in domesticated animals	None identified	N/A	N/A
Pathogen survival and spread in domesticated animals	Mandated rest days in live animal markets [86, 87]	Legislation	Success
	Mandated disinfection of livestock premises [88–90]	Legislation	Success
	Information campaign to encourage improved biosecurity practices in live animal markets [91]	Communication/marketing	Success
	Legislation around disposing of infected livestock carcasses [92, 93]	Legislation	N/A
Human exposure	Live animal market closure [89, 94–96]	Legislation	Equivocal
	Ban on trade, hunting, sale or consumption of wildlife [97–99]	Legislation	Equivocal
	Information campaign encouraging safer hunting practice [100, 101]	Communication/marketing	N/A
	Guidelines for visitor and exhibitor hand sanitation at agricultural fairs [102, 103]	Communication/marketing	N/A
	Mandated central slaughtering [104]	Legislation	N/A
Within-human barriers	Post-exposure prophylaxis (e.g. after encountering wildlife or a domesticated animal with symptoms of zoonotic disease) [105, 106]	Service provision	N/A
	Targeted vaccination of individuals with occupational exposure to animals (e.g. poultry workers) [107]	Service provision	N/A
	Mass drug administration for humans in areas of endemic disease and widespread exposure to animals [108]	Service provision	Equivocal
Multiple stages	Multi-component interventions [109–114]	Multiple approaches	Equivocal

^1^Equivocal: outcome measures varied in different directions or studies had different findings, some indicating success and some indicating failure; success: outcome measure(s) varied in the intended direction; failure: outcome measure(s) did not vary in the intended direction; N/A: no impact or outcome evaluations of this policy type identified in the review

### What are the reasons for policy success?

The evidence from the identified impact and outcome evaluations suggests that most of the policies succeeded to some extent. A range of factors contributed to policy success. First, studies emphasized the importance of effective collaboration and coordination between various agencies, disciplines, and levels of government in the execution of policy directives [114, 115], in line with a One Health approach to policy and governance. Policy success was attributed, in part, to strong working relationships that encouraged effective communication between various government agencies, and facilitated timely and appropriate policy responses [115]. Synergy between agencies responsible for surveillance and the execution of control strategies was also reported to be beneficial. For example, prompt communication and effective collaboration between laboratories testing samples and agencies implementing culls in the field was seen as important in the control of highly pathogenic avian influenza in Nigeria [116]. Similarly, authors also identified the importance of private-public relations and private sector contributions to implementing policies to prevent zoonotic spillover [112]. This included stronger government engagement with private veterinarians as a factor for success in reducing the spillover of Hendra virus in Queensland [109], and with farmers, poultry companies and national farming and poultry processing associations in Ghana as part of a successful campaign to reduce risk from highly pathogenic avian influenza [112]. Studies suggest that the inclusion of private sector stakeholders in the policy process has the potential to improve compliance through transparent dialogue around disease ecology, risk and risk mitigation [90, 91, 103, 117]; and highlight the utility of participatory approaches in prompting behaviour changes [91].

Second, authors emphasised the significance of economic incentives, suggesting that policy impact is dependent on private actors’ appraisal of costs and benefits. Studies illustrated how incentives, including compensation, subsidies, rebates, and fines, have had varying degrees of success [91, 97, 112, 115]. Compensation levels [104, 114] and enforcement practices [92] were identified as salient factors for compliance and adherence. For example, fear of sanctions for bushmeat hunting while a ban was in place in some parts of West Africa were identified as a stronger incentive to avoid bushmeat hunting than the fear of contracting Ebola virus [97]. Culls were seen as particularly challenging in this regard: while the long-term benefits for farmers may outweigh the financial loss [104], authorities need to be conscientious of the substantial economic impacts when considering policies that mandate culling or safe disposal [95]. The direct losses related to compliance (time, labour and expenses) and indirect losses due to price fluctuations and decreases in trade volume, as well as losses to associated industries, are substantial [88, 96, 113, 118].

Third, trust in government and public support for implemented policy were specified as critical factors influencing the effectiveness of disease control strategies, and research suggests that strategic engagement to facilitate compliance is a necessary step in the policy process [97]. Participatory approaches that attempt to identify and understand factors influencing compliance have been consistently used to overcome resistance to policy, as insights from engagement and consultation can lead to solutions that facilitate behaviour change at the population level [91, 103]. For example, a World Health Organization initiative to reduce avian influenza transmission in poultry markets in Indonesia worked alongside market vendors to achieve its aims, carrying out repeated consultations with the vendors and implementing market infrastructure (such as energy and running water in the market) in collaboration with local authorities to support vendor behaviour change [91].

Fourth, studies also demonstrated the importance of public communication. The quality of information, as well as the volume, complexity and delivery of public health messages, were key factors [75, 114]. Authors contend that communication strategies must understand the target audience and how they interpret and engage with messages [97], for example by building on relationships where there is exiting trust, such as between veterinarians advising animal vaccination and animal owners [117]. Homogenously delivered communication strategies were ineffectual: they limited opportunities for open discourse; discounted contradictory lived experiences and expressions of uncertainty; and ultimately contributed to scepticism surrounding implemented policies [97, 117].

Finally, studies underscored the importance of surveillance infrastructure to inform intervention strategies. Surveillance programs with the ability to collect and operationalize relevant data were essential to the development of appropriate interventions that are responsive to each unique context [115, 119]. Implementing effective surveillance programmes requires the appropriate evaluation tools [120] and trained personnel [81].

### What are the reasons for policy failure?

Studies showed that perceptions of acceptability and appropriateness were crucial to the effectiveness of implemented policies [101, 104]. Several factors were identified that negatively affected acceptability and appropriateness, including: additional expenses for private sector actors without sufficient support [75, 100, 104, 112, 114], particularly were culls were demanded but reimbursement for farmers was slow and inadequate, as in a brucellosis eradication campaign in Macedonia [81]; lack of affordable alternatives [97]; impracticality of implemented strategies [75, 101]; lack of cultural understanding in designing policy interventions [97, 100], for example the distribution of footwear to pig farmers in a Polynesian context where footwear was not traditionally worn [100]; lack of understanding of viral ecology [100]; as well as public scepticism and distrust [97, 114].

Additionally, policy ineffectiveness was associated with poor planning and execution of intervention strategies, including lack of clear direction [114]; incomplete or inconsistent implementation of control measures (17); limited scope of intervention [114]; and poor enforcement [92]. A lack of adequate resources to implement strategies also contributed to policy failure [81]. Adequate financial resources were necessary to hire and train staff to run surveillance and control operations [81]. Financial resources were also necessary to fund compensation mechanisms that facilitate compliance. Willingness to adopt policy-prescribed disposal practices was found to be associated with compensation levels (incentives) as a proportion of production price, dependency on income from activities driving zoonotic risk, and contact with prevention staff [92].

### What are the unintended consequences of implementing policies to prevent zoonotic spillover?

A small number of the included studies collected data on the unintended consequences of policies to prevent zoonotic spillover (n = 18). In some instances, unintended consequences were due to disease ecology or human behaviour as a result of policy failure. For example, a study assessing the impacts of the closure of a live poultry market found that, following the closure, vendors travelled to neighbouring markets to sell their animals [94]. As a result, while cases of avian influenza decreased in the area surrounding the closed market, cases increased in these neighbouring markets, leading to the wider geographic spread of the disease. In another study, elk were provided with supplementary feeding grounds to discourage them from coming into contact with the livestock who shared their range [65]. While this intervention had the intended consequence of reducing the transmission of brucellosis between elk and livestock, the spread of brucellosis between the elk using the supplementary feeding grounds – who were gathering in larger, tighter groups for longer periods, resulting in higher within-herd transmission – and other elk populations in the area increased. This resulted in an increasing prevalence of brucellosis among the elk, potentially increasing the risk of spillover to livestock. These examples illustrate the complexity of the social and ecological systems in which these policies are implemented, further suggesting the need for a One Health approach to policies to prevent zoonotic spillover.

A key unintended consequence can be attributed to the loss of profits and livelihoods sometimes associated with policies to prevent zoonotic spillover, as described above. The losses incurred by complying with regulations made farmers, hunters and other private sector actors reluctant to report potential infections, contributing to increased unauthorized or illegal activity, and unrestrained spread of disease [90, 92, 94, 98, 112, 114]. Studies investigated the creative ways policy enforcement was circumvented, including hiding hunting equipment on the outskirts of towns or developing informal trade markets and networks [97, 98]. Unintended consequences identified in the included evaluations emphasize an opportunity for policymakers to improve sector compliance through public education, levying the influence of consumer attitudes on industry standards [104, 113].

### How has evaluation of these policies been approached in the literature?

A range of study designs were used to evaluate policies. Outcome evaluations (n = 33) used time series or repeat cross-sectional data to conduct evaluations of natural experiments, though most studies did not include a control group for comparison. Outcome evaluations also used case-control and modelling approaches to assess policy impact on an outcome of interest. Process evaluations (n = 30) used cross-sectional and qualitative approaches, as well as study designs combining multiple sources of data, to understand aspects of policy implementation such as the extent to which the policy was being implemented as designed, and the responses and attitudes of stakeholders involved in policy implementation. Economic evaluations (n = 11) included cost-benefit analyses, risk-benefit analyses and modelling studies. Formative evaluations (n = 17) used modelling approaches to estimate what the impacts of a proposed policy option would be in a specific context.

Outcome variables interpreted as indicators of policy success were also numerous and represented determinants along the spillover pathway. As expected, many studies assessed impact on disease transmission, including disease prevalence and incidence, disease eradication, case numbers, and basic reproduction number in human and animal populations, as well as evidence of disease in environmental samples, such as in live animal markets or at carcass disposal sites. Studies also assessed impacts on intermediate factors indicative of successful implementation of specific policies, such as the availability of wild species in markets where a trade ban had been implemented, or knowledge and practices of stakeholders in response to an educational or information campaign.

While most studies found a reduced risk of zoonotic spillover following policy implementation, comparing the magnitude of these impacts was challenging due to the variety of study designs and outcome measures used in the included studies. However, we identified several studies which used modelling to directly compare the impacts of policy options. These studies evaluated various policy scenarios: different combinations within multi-component policy interventions [121]; culling versus vaccinating wildlife [122] and livestock [84, 85] populations; targeting strategies to humans exclusively versus targeting humans and livestock [108]; and altering the parameters for culling and vaccination strategies, for example by modelling different ranges for culling and vaccination near infected farms [85]. These studies often highlighted trade-offs between the effectiveness of policy measures and their cost. For example, estimates of the number of infected flocks were lower when incorporating a ring cull (cull of animals on farms surrounding an outbreak) into a multi-component control strategy for highly pathogenic avian influenza [121]. However, livestock vaccination was estimated to be a highly effective strategy, with one study findings livestock vaccination to be as or more effective than a pre-emptive cull for outbreak control purposes (depending on the extent of vaccination coverage), while minimising the number of animals culled [85]. One study jointly modelled costs and benefits of strategies, and found that livestock vaccination had a higher cost-benefit ratio than a wildlife cull [122]. A final study highlighted the potential of holistic approaches, with drug administration in humans and livestock having a lower cost per disability-adjusted life year averted than intervention in humans alone [108].

Study authors noted a number of challenges encountered while evaluating policies to prevent zoonotic spillover. One study noted the difficulty of determining the impact of policies aiming to reduce spillover events between wildlife, livestock and humans, as the number of spillover events is often relatively small [65]. This highlights the importance of considering upstream determinants and risk factors as outcome measures in attempting to evaluate these policies, particularly where spillover events may happen infrequently or not at all during the period of observation. Studying changes in risk factors for spillover can provide insight on the effectiveness of different policies in tackling spillover risk.

Lack of suitable data was a frequently cited barrier to policy evaluation. As policies to prevent zoonotic spillover are often reactive, being implemented in response to an outbreak in animal populations, accessing data from before a policy was implemented was challenging. Studies highlighted the value of routinely collected data, which was often the only data available and was frequently used for policy evaluation [65, 66, 94, 115, 119, 123]. However, in many contexts routine data on animal health is not collected [80]. Routine testing data from livestock can sometimes be used for evaluation where it exists, but it does not always provide sufficient detail for examining the potential for a policy to prevent zoonotic spillover. For example, some tests do not differentiate between current and past infection, making it difficult to identify where and when spillover occurred [65], and animal health data may not be granular enough for policy evaluation, particularly in terms of evaluating local policies [94]. Studies also highlighted instances where the private sector may own data sets reporting disease prevalence and transmission, but may be reluctant to share the data for evaluation purposes [121]. In such instances, open communication and good relationships with the private sector may be facilitators to evaluation.

Beyond the lack of baseline data, studies highlighted the difficulty in collecting information about policy compliance. As failing to comply often puts farmers and hunters at risk of fines or imprisonment, they were reluctant to disclose information about non-compliance or participation in illegal trade and sale of animals [86, 92, 97, 112]. This made it difficult to determine policy effectiveness.

### Quality assessment

Of the 44 quantitative evaluations, 37 were evaluated as being at moderate or higher risk of bias (see Supplementary File 4), given the possibility of bias in the assessment of intervention impact due to the presence of confounding effects. A small number of studies were determined to be at serious (n = 6) or critical (n = 1) risk of bias, for two main reasons: only having data from after the intervention was implemented; or using a case-control study model without measuring and adjusting for important potential confounders, such as the prevalence of a targeted disease prior to policy implementation. These limitations may reflect the nature of zoonotic spillover events and policy responses, which can happen quickly and leave little time for baseline data collection. Many of the included studies relied on surveillance data, but where such data sets are not available, post-test and case-control study designs may be the only options.

The quality of studies assessed with the tool developed based on Dixon-Woods’ approach [55] was high overall (n = 41, see Supplementary file 5). Most studies were rated as high in terms of clearly and comprehensively presenting their results (n = 37), analysis (n = 34), research design (n = 33), aims (n = 32) and research process (n = 28). Most studies also had a high relevance to the research question (n = 31), indicating that the research was embedded in policy, being commissioned, co-designed or conducted in partnership with government stakeholders.

## Discussion

We identified a range of policies targeting different parts of the spillover pathway implemented by various policy and governance sectors, including some multi-sectoral initiatives. Policies tended to rely heavily on private sector actors (including actors ranging from small-scale farmers and hunters to larger commercial operations) for implementation, suggesting that open communication and collaboration with these actors was essential for successful policy implementation. Policy success was undermined by lack of collaboration between government agencies; lack of communication between surveillance and control operations; poor understanding of the context in which policies were implemented; and inadequate financial compensation for private sector actors who lost profits and incurred additional costs by complying with policies. Where policies were ineffective, this tended to be due to unintended consequences relating to complex dynamics within the social and ecological systems where policies were implemented. Lack of appropriate data was a key obstacle to policy evaluation, and studies emphasised the importance of robust surveillance infrastructure in evaluating policies that tended to be implemented reactively, in response to an outbreak of zoonotic disease in animal or human populations.

### Implications for policy and practice

The key role that the private sector and industry actors play in implementing policies to prevent zoonotic spillover is an important consideration for policymakers. Our findings suggest that many of these policies must be complied with by farmers – from subsistence and smallholder farmers to large corporations – as well as by other actors, such as hunters. Lack of awareness as well as financial costs of compliance among these groups present key barriers to policy success in this area. This set of stakeholders is complex as some may make very marginal profits, if any, and may struggle to afford the additional costs of implementing preventive policies. However, powerful actors and profitable industries are also involved, including large-scale farms and primary resource extraction enterprises [22]. Acknowledging the differences across these stakeholder groups, and in particular assessing their capacity to bear some of the costs related to prevention, emerges as crucial in successful policy implementation.

Finally, our findings highlight the importance of disease surveillance in efforts to reduce the risk of spillover events. As well as acting as an early warning system, surveillance provides a source of data to evaluate the impact of preventive policies. We found the availability of surveillance data to be a key enabling factor in evaluating policies. In addition, close collaboration between agencies responsible for disease surveillance and control efforts was key to policy success. National surveillance efforts, as well as cross-country collaboration to support global efforts, such as the United States Agency for International Development’s PREDICT program supporting surveillance in areas at high risk for zoonotic disease outbreaks [124], must be sustained and expanded. In complex areas such as the prevention of zoonotic spillover, approaches to surveillance which encompass risk factors and transmission pathways [125], as well as One Health surveillance systems which harmonise and integrate data collection and analysis from across human, animal and environmental sectors [126], are promising approaches to developing surveillance systems that support risk. This context also involves a need to strengthen surveillance capacity in remote and rural locations, as communities living in these contexts may have exposure to numerous pathogens of wildlife origin. This will require strengthening clinical and diagnostic capacity in these settings, as well as engaging with stakeholders such as community human and animal health workers and wildlife or national park rangers [127].

### Comparison with existing literature

This review sought to map the range of policies implemented to reduce the risk of zoonotic spillover, and the various approaches taken to evaluation, and identify factors behind the success and failure of policy implementation and evaluation. Due to this broad scope, comparing relative effectiveness of policy interventions was challenging. Existing systematic reviews with a more specific focus could apply meta-analysis to determine which interventions were most effective. For example, a review of market-level biosecurity measures aiming to reduce the transmission of avian influenza found that reducing market size, separating poultry species, cleaning and disinfecting premises, closing markets and banning overnight storage were highly effective interventions [45]. However, our findings suggest that studies focused on the control of avian influenza dominate the literature in this space (55 out of 111 evaluated policies), and many of these are focused on market-level measures. Systematic reviews focused on other approaches to reduce spillover risk, such as on-farm biosecurity [47]; biosecurity for backyard poultry rearing [46]; and community-based interventions [28] comment on the paucity of high-quality evidence around the impacts of such approaches. By taking a broad perspective, we hope our findings will provide policy options for consideration in a number of contexts, and guide researchers in focusing their efforts on areas where evidence is lacking.

### Strengths and weaknesses of the study

To our knowledge, this is the first attempt to systematically identify and document evaluations of policies aiming to prevent the spillover of zoonotic pathogens into human populations. However, because of the complex drivers of spillover events, some potentially relevant policy evaluations may be excluded where their outcome measures are too far removed from zoonotic spillover. While relevant, such evaluations will be difficult to systematically identify as they make no reference to zoonotic disease.

In addition, this review focused on policy evaluations that have been reported in the peer-reviewed literature and the grey literature published by international agencies and organisations working on these topics. Policies that have been implemented but not evaluated, or evaluated but not published in these literatures, will therefore be excluded from this review. As a result, potentially effective and important policies in the prevention of zoonotic spillover events may not have been identified. However, we hope that the findings from this review will highlight these gaps in the evaluative evidence. We also hope that this review, by extracting practical dimensions, such as study design, outcome measures and the challenges encountered in the evaluation process, will support policymakers and researchers in carrying out further policy evaluations in this space.

#### Unanswered questions and future research

Our findings highlight several important gaps in the evidence. First, while observational evidence emphasises the importance of upstream determinants such as environmental and ecosystem health in the increasing rate of zoonotic spillover [1, 15], we only identified a single evaluation of a policy attempting to target one of these upstream determinants: an evaluation carried out in China to assess the impact of the Ramstar wetland protection program on avian influenza in migratory waterfowl [66]. This study found that proximity to protected wetlands reduced outbreak risk. Authors hypothesised that this effect was due to the separation of wild waterfowl and poultry populations and the diversion of wild waterfowl away from human-dominated landscapes and toward protected natural habitats. Our findings support existing calls for more quantitative and mechanistic studies of the impact of interventions supporting environmental and ecosystem health on zoonotic spillover risk [128], as well as calls for greater integration of the environment into One Health research, policy and practice [31]. Further evaluations of environment and habitat protection policies would strengthen our understanding of this area. In addition, the impact of policies to reduce deforestation or expand forest coverage, such as China’s Grain-to-Green program [129], on the spillover pathway could be evaluated. Such evaluations might consider potential unintended consequences, as these policies could promote healthier wildlife populations with better disease resistance, but may also facilitate wildlife population growth and higher rates of wildlife-human encounters [130].

There is also a lack of evaluation of policies targeting infection intensity and pathogen release in either wildlife or domesticated animals. These could include approaches such as improving animal health and welfare to make these populations more resistant to disease [13]. While arguments have been made for strengthening legal structures supporting animal welfare in order to reduce the risk of zoonotic pathogen transmission [131], there is a need to evaluate policies that take this approach.

## Conclusion

Our review found publications evaluating a wide range of policy interventions spanning the spillover pathway, including habitat protection; trade regulations; border control and quarantine procedures; farm and market biosecurity measures; public information campaigns; and vaccination programmes for wildlife and domesticated animals, as well as human populations with occupational exposure to animals. A wide range of governance sectors implemented these policies, highlighting the prevention of zoonotic spillover as a cross-sectoral issue, though most policies were implemented by a single sector. Our findings highlight the importance of industry and private actors in implementing policies to prevent zoonotic spillover, and the need for thoughtful and effective engagement with this wide range of actors, from subsistence hunters and farmers through to industrial animal agriculture operations to address their concerns through a range of incentives. We also identified the centrality of surveillance data in evaluating policies that are often implemented reactively, and effective collaboration between surveillance and control operations as a central factor in successful policy implementation.

### Electronic supplementary material

Below is the link to the electronic supplementary material.

Supplementary Material 1

Supplementary Material 2

Supplementary Material 3

Supplementary Material 4

Supplementary Material 5

## Data Availability

All data generated or analysed during this study are included in this published article and its supplementary information files. Analysis code for descriptive characteristics of included policies is available on GitHub.

## List of abbreviations

EID: Emerging infectious disease

## References

[1] Morse SS, Mazet JA, Woolhouse M, Parrish CR, Carroll D, Karesh WB, Zambrana-Torrelio C, Lipkin WI, Daszak P (2012). Prediction and prevention of the next pandemic zoonosis. The Lancet.

[2] Pulliam JRC, Epstein JH, Dushoff J, Rahman SA, Bunning M, Jamaluddin AA, Hyatt AD, Field HE, Dobson AP, Daszak P (2012). Agricultural intensification, priming for persistence and the emergence of Nipah virus: a lethal bat-borne zoonosis. J Royal Soc Interface.

[3] IPCC. In: Pörtner H-O, Roberts DC, Tignor M, Poloczanska ES, Mintenbeck K, Alegría A, Craig M, Langsdorf S, Löschke S, Möller V, Okem A, Rama B, editors. Climate Change 2022: impacts, adaptation and vulnerability, contribution of Working Group II to the Sixth Assessment Report of the Intergovernmental Panel on Climate Change. In press ed. Cambridge University Press; 2022.

[4] Brenner N, Ghosh S (2022). Between the colossal and the catastrophic: planetary urbanization and the political ecologies of emergent Infectious Disease. Environ Plan A.

[5] Gallo-Cajiao E, Lieberman S, Dolšak N (2023). Global governance for pandemic prevention and the wildlife trade. Lancet Planet Health.

[6] Marco MD, Baker ML, Daszak P (2020). Opinion: sustainable development must account for pandemic risk. PNAS.

[7] Heymann DL, Dixon M, Mackenzie JS, Jeggo M, Daszak P, Richt JA (2013). Infections at the Animal/Human interface: shifting the paradigm from emergency response to Prevention at source. One health: the human-animal-environment interfaces in Emerging Infectious Diseases: Food Safety and Security, and International and National plans for implementation of one health activities.

[8] United Nations Environment Programme, International Livestock Research Institute. (2020) Preventing the next pandemic: Zoonotic diseases and how to break the chain of transmission. 82.

[9] Intergovernmental Science-Policy Platform On Biodiversity And Ecosystem Services (IPBES). (2020) Workshop Report on Biodiversity and Pandemics of the Intergovernmental Platform on Biodiversity and Ecosystem Services (IPBES). 10.5281/ZENODO.4147317.

[10] One Health theory of change. https://www.who.int/publications/m/item/one-health-theory-of-change. Accessed 30 Jan 2023.

[11] Vinuales J, Moon S, Moli GL, Burci G-L (2021). A global pandemic treaty should aim for deep prevention. The Lancet.

[12] Plowright RK, Parrish CR, McCallum H, Hudson PJ, Ko AI, Graham AL, Lloyd-Smith JO (2017). Pathways to zoonotic spillover. Nat Rev Microbiol.

[13] Sokolow SH, Nova N, Pepin KM (2019). Ecological interventions to prevent and manage zoonotic pathogen spillover. Philosophical Trans Royal Soc B: Biol Sci.

[14] Johnson CK, Hitchens PL, Pandit PS, Rushmore J, Evans TS, Young CCW, Doyle MM (2020). Global shifts in mammalian population trends reveal key predictors of virus spillover risk. Proc Royal Soc B: Biol Sci.

[15] Allen T, Murray KA, Zambrana-Torrelio C, Morse SS, Rondinini C, Di Marco M, Breit N, Olival KJ, Daszak P (2017). Global hotspots and correlates of emerging zoonotic Diseases. Nat Commun.

[16] Gandy M (2022). THE ZOONOTIC CITY: Urban Political Ecology and the pandemic imaginary. Int J Urban Reg Res.

[17] Hardi R, Babocsay G, Tappe D, Sulyok M, Bodó I, Rózsa L (2017). Armillifer-infected snakes sold at Congolese Bushmeat Markets Represent an emerging zoonotic threat. EcoHealth.

[18] Steve A-M, Ahidjo A, Placide M-K (2017). High prevalences and a wide genetic diversity of Simian Retroviruses in non-human Primate Bushmeat in Rural areas of the Democratic Republic of Congo. EcoHealth.

[19] Weiss S, Nowak K, Fahr J, Wibbelt G, Mombouli J-V, Parra H-J, Wolfe ND, Schneider BS, Leendertz FH (2012). Henipavirus-related sequences in Fruit Bat Bushmeat, Republic of Congo. Emerg Infect Dis.

[20] Aguirre AA, Catherina R, Frye H, Shelley L (2020). Illicit Wildlife Trade, Wet Markets, and COVID-19: preventing future pandemics. World Med Health Policy.

[21] Nadimpalli ML, Pickering AJ (2020). A call for global monitoring of WASH in wet markets. Lancet Planet Health.

[22] Viliani F, Edelstein M, Buckley E, Llamas A, Dar O (2017). Mining and emerging infectious Diseases: results of the Infectious Disease Risk Assessment and Management (IDRAM) initiative pilot. The Extractive Industries and Society.

[23] Wegner GI, Murray KA, Springmann M, Muller A, Sokolow SH, Saylors K, Morens DM (2022). Averting wildlife-borne Infectious Disease epidemics requires a focus on socio-ecological drivers and a redesign of the global food system. eClinicalMedicine.

[24] Daszak P (2012). Anatomy of a pandemic. The Lancet.

[25] Joint Tripartite (FAO, OIE, WHO) and UNEP Statement. Tripartite and UNEP support OHHLEP’s definition of one health. ” OIE - World Organisation for Animal Health; 2021.

[26] (2022) One Health Joint Plan of Action, 2022–2026. 10.4060/cc2289en.

[27] Baum SE, Machalaba C, Daszak P, Salerno RH, Karesh WB (2017). Evaluating one health: are we demonstrating effectiveness?. One Health.

[28] Halton K, Sarna M, Barnett A, Leonardo L, Graves N (2013). A systematic review of community-based interventions for emerging zoonotic infectious Diseases in Southeast Asia. JBI Database System Rev Implement Rep.

[29] Meyer A, Holt HR, Selby R, Guitian J (2016). Past and Ongoing Tsetse and Animal Trypanosomiasis Control Operations in five African countries: a systematic review. PLoS Negl Trop Dis.

[30] Howlett M, Cashore B, Engeli I, Allison CR (2014). Conceptualizing Public Policy. Comparative Policy studies: conceptual and methodological challenges.

[31] Barrett MA, Bouley TA (2015). Need for enhanced environmental representation in the implementation of one health. EcoHealth.

[32] Barbrook-Johnson P, Proctor A, Giorgi S, Phillipson J (2020). How do policy evaluators understand complexity?. Evaluation.

[33] Saunders-Hastings P, Crispo JAG, Sikora L, Krewski D (2017). Effectiveness of personal protective measures in reducing pandemic Influenza transmission: a systematic review and meta-analysis. Epidemics.

[34] Bin Nafisah S, Alamery AH, Al Nafesa A, Aleid B, Brazanji NA (2018). School closure during novel Influenza: a systematic review. J Infect Public Health.

[35] Viner RM, Russell SJ, Croker H, Packer J, Ward J, Stansfield C, Mytton O, Bonell C, Booy R (2020). School closure and management practices during coronavirus outbreaks including COVID-19: a rapid systematic review. The Lancet Child & Adolescent Health.

[36] Juneau C-E, Pueyo T, Bell M, Gee G, Collazzo P, Potvin L. (2020) Evidence-Based, cost-effective interventions to suppress the COVID-19 pandemic: a systematic review. medRxiv 2020.04.20.20054726. 10.1186/s13643-022-01958-9 PMC9096744 35550674

[37] MacIntyre CR, Chughtai AA (2015). Facemasks for the prevention of Infection in healthcare and community settings. BMJ.

[38] Smith SMS, Sonego S, Wallen GR, Waterer G, Cheng AC, Thompson P (2015). Use of non-pharmaceutical interventions to reduce the transmission of Influenza in adults: a systematic review. Respirology.

[39] Jefferson T, Del Mar CB, Dooley L et al. (2011) Physical interventions to interrupt or reduce the spread of respiratory viruses. Cochrane Database Syst Rev CD006207. 10.1002/14651858.CD006207.pub4 PMC6993921 21735402

[40] Astbury CC, Lee KM, Aguiar R (2022). Policies to prevent zoonotic spillover: protocol for a systematic scoping review of evaluative evidence. BMJ Open.

[41] Tricco AC, Lillie E, Zarin W (2018). PRISMA Extension for scoping reviews (PRISMA-ScR): Checklist and Explanation. Ann Intern Med.

[42] Arksey H, O’Malley L (2005). Scoping studies: towards a methodological framework. Int J Soc Res Methodol.

[43] Levac D, Colquhoun H, O’Brien KK (2010). Scoping studies: advancing the methodology. Implement Sci.

[44] Colquhoun HL, Levac D, O’Brien KK, Straus S, Tricco AC, Perrier L, Kastner M, Moher D (2014). Scoping reviews: time for clarity in definition, methods, and reporting. J Clin Epidemiol.

[45] Zhou X, Wang Y, Liu H, Guo F, Doi SA, Smith C, Clements ACA, Edwards J, Huang B, Soares Magalhães RJ (2018). Effectiveness of Market-Level Biosecurity at reducing exposure of Poultry and humans to Avian Influenza: a systematic review and Meta-analysis. J Infect Dis.

[46] Conan A, Goutard FL, Sorn S, Vong S (2012). Biosecurity measures for backyard poultry in developing countries: a systematic review. BMC Vet Res.

[47] Youssef DM, Wieland B, Knight GM, Lines J, Naylor NR (2021). The effectiveness of biosecurity interventions in reducing the transmission of bacteria from livestock to humans at the farm level: a systematic literature review. Zoonoses Public Health.

[48] Shi N, Huang J, Zhang X, Bao C, Yue N, Wang Q, Cui T, Zheng M, Huo X, Jin H (2020). Interventions in live poultry markets for the Control of Avian Influenza: a systematic review and Meta-analysis. J Infect Dis.

[49] Cupertino MC, Resende MB, Mayer NA, Carvalho LM, Siqueira-Batista R (2020). Emerging and re-emerging human infectious Diseases: a systematic review of the role of wild animals with a focus on public health impact. Asian Pac J Trop Med.

[50] Clifford Astbury C, Demeshko A, McLeod R, Wiktorowicz M, Gallo Caijao E, Cullerton K, Lee KM, Viens AM, Penney TL. (2023) Governance of the wildlife trade and prevention of emerging zoonoses: a mixed methods network analysis of global organisations. [In preparation]. 10.1186/s12992-024-01055-7 PMC11188226 38902738

[51] Covidence - Better. systematic review management. https://www.covidence.org/home. Accessed 17 Jul 2020.

[52] Effective Public Health Practice Project. (2009) Quality Assessment Tool for Quantitative Studies. 4.

[53] Sterne JA, Hernán MA, Reeves BC (2016). ROBINS-I: a tool for assessing risk of bias in non-randomised studies of interventions. BMJ.

[54] Clifford Astbury C, McGill E, Egan M, Penney TL (2021). Systems thinking and complexity science methods and the policy process in non-communicable Disease prevention: a systematic scoping review protocol. BMJ Open.

[55] Dixon-Woods M, Cavers D, Agarwal S (2006). Conducting a critical interpretive synthesis of the literature on access to healthcare by vulnerable groups. BMC Med Res Methodol.

[56] Braun V, Clarke V (2006). Using thematic analysis in psychology. Qualitative Res Psychol.

[57] (2021) Dedoose Version 8.3.47, web application for managing, analyzing, and presenting qualitative and mixed method research data.

[58] Page MJ, McKenzie JE, Bossuyt PM (2021). The PRISMA 2020 statement: an updated guideline for reporting systematic reviews. BMJ.

[59] Park H, Chun MS, Joo Y (2020). Traumatic stress of frontline workers in culling livestock animals in South Korea. Animals.

[60] Programme UNE. Effectiveness of policy interventions relating to the illegal and unsustainable. Wildlife Trade - Policy Brief; 2019.

[61] Cito F, Narcisi V, Danzetta ML, Iannetti S, Sabatino DD, Bruno R, Carvelli A, Atzeni M, Sauro F, Calistri P (2013). Analysis of Surveillance systems in Place in European Mediterranean Countries for West Nile Virus (WNV) and Rift Valley Fever (RVF). Transbound Emerg Dis.

[62] Schwind JS, Goldstein T, Thomas K, Mazet JA, Smith WA, PREDICT Consortium (2014). Capacity building efforts and perceptions for wildlife surveillance to detect zoonotic pathogens: comparing stakeholder perspectives. BMC Public Health.

[63] Reisen WK, Kramer VL, Barker CM (2003). CALIFORNIA STATE MOSQUITO-BORNE VIRUS SURVEILLANCE AND RESPONSE PLAN: A RETROSPECTIVE EVALUATION USING CONDITIONAL SIMULATIONS *. Am J Trop Med Hyg.

[64] Smith GC, Cheeseman CL (2002). A mathematical model for the control of Diseases in wildlife populations: culling, vaccination and fertility control. Ecol Model.

[65] Brennan A, Cross PC, Portacci K, Scurlock BM, Edwards WH (2017). Shifting brucellosis risk in livestock coincides with spreading seroprevalence in elk. PLoS ONE.

[66] Wu T, Perrings C, Shang C, Collins JP, Daszak P, Kinzig A, Minteer BA (2020). Protection of wetlands as a strategy for reducing the spread of avian Influenza from migratory waterfowl. Ambio.

[67] Basinski AJ, Nuismer SL, Remien CH (2019). A little goes a long way: weak vaccine transmission facilitates oral vaccination campaigns against zoonotic pathogens. PLoS Negl Trop Dis.

[68] Selhorst T. (1999) An evaluation of the efficiency of rabies control strategies in fox (Vulpes 6ulpes) populations using a computer simulation program. Ecol Model 12.

[69] Shwiff SA, Sterner RT, Hale R, Jay MT, Sun B, Slate D (2009). Benefit cost scenarios of potential oral rabies vaccination for Skunks in California. J Wildl Dis.

[70] García-Díaz P, Ross JV, Woolnough AP, Cassey P (2017). Managing the risk of wildlife Disease introduction: pathway‐level biosecurity for preventing the introduction of alien ranaviruses. J Appl Ecol.

[71] Hassim A, Dekker EH, Byaruhanga C, Reardon T, van Heerden H (2017). A retrospective study of anthrax on the Ghaap Plateau, Northern Cape province of South Africa, with special reference to the 2007–2008 outbreaks. Onderstepoort J Vet Res.

[72] Knight-Jones TJD, Gibbens J, Wooldridge M, Staerk KDC (2011). Assessment of Farm-Level Biosecurity Measures after an outbreak of Avian Influenza in the United Kingdom. Transbound Emerg Dis.

[73] Karabozhilova I, Wieland B, Alonso S, Salonen L, Häsler B (2012). Backyard chicken keeping in the Greater London Urban Area: welfare status, biosecurity and Disease control issues. Br Poult Sci.

[74] Manyweathers J, Field H, Jordan D, Longnecker N, Agho K, Smith C, Taylor M (2017). Risk mitigation of emerging zoonoses: Hendra Virus and Non-vaccinating Horse Owners. Transbound Emerg Dis.

[75] Kung N, McLaughlin A, Taylor M, Moloney B, Wright T, Field H (2013). Hendra virus and horse owners - risk perception and management. PLoS ONE.

[76] Rasouli J, Holakoui K, Forouzanfar MH, Salari S, Bahoner, Rashidian A (2009). Cost effectiveness of livestock vaccination for brucellosis in West-Azerbayjan province. Urmia Med J.

[77] El Masry I, Rijks J, Peyre M, Taylor N, Lubroth J, Jobre Y (2014). Modelling Influenza A H5N1 vaccination strategy scenarios in the household poultry sector in Egypt. Trop Anim Health Prod.

[78] Mroz C, Gwida M, El-Ashker M, Ziegler U, Homeier-Bachmann T, Eiden M, Groschup MH (2017). Rift valley Fever virus Infections in Egyptian cattle and their prevention. Transbound Emerg Dis.

[79] Pinsent A, Pepin KM, Zhu H, Guan Y, White MT, Riley S. (2017) The persistence of multiple strains of avian influenza in live bird markets. Proceedings of the Royal Society B: Biological Sciences 284:20170715. 10.1098/rspb.2017.0715 PMC5740266 29212718

[80] Abbas B, Yousif MA, Nur HM (2014). Animal health constraints to livestock exports from the Horn of Africa: -EN- -FR- restrictions sanitaires imposées aux exportations de bétail à partir de la corne de l’Afrique -ES- limitaciones zoosanitarias a las exportaciones de ganado desde El Cuerno De África. Rev Sci Tech OIE.

[81] Naletoski I, Kirandziski T, Mitrov D, Krstevski K, Dzadzovski I, Acevski S (2010). Gaps in brucellosis eradication campaign in Sheep and goats in Republic of Macedonia: lessons learned. Croat Med J.

[82] Weaver JT, Malladi S, Bonney PJ, Patyk KA, Bergeron JG, Middleton JL, Alexander CY, Goldsmith TJ, Halvorson DA (2016). A Simulation-based evaluation of Premovement active surveillance Protocol options for the Managed Movement of Turkeys to Slaughter during an outbreak of highly pathogenic avian Influenza in the United States. Avian Dis.

[83] Andronico A, Courcoul A, Bronner A, Scoizec A, Lebouquin-Leneveu S, Guinat C, Paul MC, Durand B, Cauchemez S (2019). Highly pathogenic avian Influenza H5N8 in south-west France 2016–2017: a modeling study of control strategies. Epidemics.

[84] Backer JA, van Roermund HJW, Fischer EAJ, van Asseldonk MAPM, Bergevoet RHM (2015). Controlling highly pathogenic avian Influenza outbreaks: an epidemiological and economic model analysis. Prev Vet Med.

[85] Backer JA, Hagenaars TJ, van Roermund HJW, de Jong MCM (2009). Modelling the effectiveness and risks of vaccination strategies to control classical swine Fever epidemics. J R Soc Interface.

[86] Fournie G, Guitian FJ, Mangtani P, Ghani AC (2011). Impact of the implementation of rest days in live bird markets on the dynamics of H5N1 highly pathogenic avian Influenza. J R Soc Interface.

[87] Kung NY, Guan Y, Perkins NR, Bissett L, Ellis T, Sims L, Morris RS, Shortridge KF, Peiris JSM (2003). The impact of a monthly Rest Day on Avian Influenza Virus isolation rates in Retail Live Poultry markets in Hong Kong. Avian Dis.

[88] Horigan V, Gale P, Adkin A, Brown I, Clark J, Kelly L (2019). A qualitative risk assessment of cleansing and disinfection requirements after an avian Influenza outbreak in commercial poultry. Br Poult Sci.

[89] Yuan J, Lau EHY, Li K (2015). Effect of live Poultry Market Closure on Avian Influenza A(H7N9) virus activity in Guangzhou, China, 2014. Emerg Infect Dis.

[90] Fournie G, Guitian J, Desvaux S, Cuong VC, Dung DH, Pfeiffer DU, Mangtani P, Ghani AC (2013). Interventions for avian Influenza A (H5N1) risk management in live bird market networks. Proc Natl Acad Sci USA.

[91] Samaan G, Hendrawati F, Taylor T, Pitona T, Marmansari D, Rahman R, Lokuge K, Kelly PM (2012). Application of a healthy food markets guide to two Indonesian markets to reduce transmission of avian Flu. Bull World Health Organ.

[92] Huang Z, Wang J, Zuo A (2017). Chinese farmers’ willingness to accept compensation to practice safe disposal of HPAI infected chicken. Prev Vet Med.

[93] Graiver DA, Topliff CL, Kelling CL, Bartelt-Hunt SL (2009). Survival of the avian Influenza virus (H6N2) after land disposal. Environ Sci Technol.

[94] Li Y, Wang Y, Shen C, Huang J, Kang J, Huang B, Guo F, Edwards J (2018). Closure of live bird markets leads to the spread of H7N9 Influenza in China. PLoS ONE.

[95] Ma J, Yang N, Gu H, Bai L, Sun J, Gu S, Gu J (2019). Effect of closure of live poultry markets in China on prevention and control of human Infection with H7N9 avian Influenza: a case study of four cities in Jiangsu Province. J Public Health Policy.

[96] Chen Y, Cheng J, Xu Z, Hu W, Lu J (2020). Live poultry market closure and avian Influenza A (H7N9) Infection in cities of China, 2013–2017: an ecological study. BMC Infect Dis.

[97] Bonwitt J, Dawson M, Kandeh M, Ansumana R, Sahr F, Brown H, Kelly AH (2018). Unintended consequences of the `bushmeat ban’ in West Africa during the 2013–2016 Ebola virus Disease epidemic. Soc Sci Med.

[98] Brooks-Moizer F, Roberton SI, Edmunds K, Bell D (2009). Avian Influenza H5N1 and the wild Bird Trade in Hanoi, Vietnam. Ecol Soc.

[99] Cardador L, Tella JL, Anadon JD, Abellan P, Carrete M (2019). The European trade ban on wild birds reduced invasion risks. Conserv Lett.

[100] Guerrier G, Foster H, Metge O, Chouvin C, Tui M (2013). Cultural contexts of swine-related Infections in Polynesia. Clin Microbiol Infect.

[101] Massey PD, Polkinghorne BG, Durrheim DN, Lower T, Speare R (2011). Blood, guts and knife cuts: reducing the risk of swine brucellosis in feral pig hunters in north-west New South Wales, Australia. Rural Remote Health.

[102] Lauterbach SE, Nelson SW, Martin AM, Spurck MM, Mathys DA, Mollenkopf DF, Nolting JM, Wittum TE, Bowman AS. (2020) Adoption of recommended hand hygiene practices to limit zoonotic Disease transmission at agricultural fairs. Preventive Veterinary Medicine. 10.1016/j.prevetmed.2020.105116. 10.1016/j.prevetmed.2020.105116 PMC7494593 32768662

[103] Stewart RJ, Rossow J, Conover JT (2018). Do animal exhibitors support and follow recommendations to prevent transmission of variant Influenza at agricultural fairs? A survey of animal exhibitor households after a variant Influenza virus outbreak in Michigan. Zoonoses Public Health.

[104] Lin X, Zhang D, Wang X, Huang Y, Du Z, Zou Y, Lu J, Hao Y (2017). Attitudes of consumers and live-poultry workers to central slaughtering in controlling H7N9: a cross-sectional study. BMC Public Health.

[105] Huot C, De Serres G, Duval B, Maranda-Aubut R, Ouakki M, Skowronski DM (2008). The cost of preventing rabies at any cost: post-exposure prophylaxis for occult bat contact. Vaccine.

[106] De Serres G, Skowronski DM, Mimault P, Ouakki M, Maranda-Aubut R, Duval B (2009). Bats in the bedroom, bats in the Belfry: reanalysis of the rationale for rabies postexposure Prophylaxis. Clin Infect Dis.

[107] Vivancos R, Showell D, Keeble B, Goh S, Kroese M, Lipp A, Battersby J (2011). Vaccination of Poultry workers: delivery and uptake of Seasonal Influenza immunization. Zoonoses Public Health.

[108] Okello AL, Thomas LF (2017). Human taeniasis: current insights into prevention and management strategies in endemic countries. RISK MANAG HEALTHC POLICY.

[109] Mendez D, Buttner P, Speare R (2014). Hendra virus in Queensland, Australia, during the winter of 2011: veterinarians on the path to better management strategies. Prev Vet Med.

[110] Häsler B, Howe KS, Hauser R, Stärk KDC (2012). A qualitative approach to measure the effectiveness of active avian Influenza virus surveillance with respect to its cost: a case study from Switzerland. Prev Vet Med.

[111] Brinkley C, Kingsley JS, Mench J (2018). A Method for Guarding Animal Welfare and Public Health: tracking the rise of Backyard Poultry ordinances. J Community Health.

[112] Turkson PK, Okike I (2016). Assessment of practices, capacities and incentives of poultry chain actors in implementation of highly pathogenic avian Influenza mitigation measures in Ghana. Vet Med Sci.

[113] Akunzule AN, Koney EBM, Tiongco M (2009). Economic impact assessment of highly pathogenic avian Influenza on the poultry industry in Ghana. Worlds Poult Sci J.

[114] Hunter C, Birden HH, Toribio J-A, Booy R, Abdurrahman M, Ambarawati AIGAA, Adiputra N. (2014) Community preparedness for highly pathogenic avian Influenza on Bali and Lombok, Indonesia. Rural Remote Health 14. 25224284

[115] Tustin J, Laberge K, Michel P (2011). A National Epidemic of Campylobacteriosis in Iceland, lessons learned. Zoonoses Public Health.

[116] Oladokun AT, Meseko CA, Ighodalo E, John B. Ekong PS Effect of intervention on the control of highly pathogenic avian Influenza in Nigeria. 8. PMC3527058 23308319

[117] Manyweathers J, Field H, Longnecker N, Agho K, Smith C, Taylor M (2017). Why won’t they just vaccinate? Horse owner risk perception and uptake of the Hendra virus vaccine. BMC Vet Res.

[118] Zhu G, Kang M, Wei X, Tang T, Liu T, Xiao J, Song T, Ma W (2020). Different intervention strategies toward live poultry markets against avian Influenza A (H7N9) virus: model-based assessment. Environ Res.

[119] Chowell G, Simonsen L, Towers S, Miller MA, Viboud C (2013). Transmission potential of Influenza A/H7N9, February to May 2013, China. BMC Med.

[120] Bodenham RF, Mtui-Malamsha N, Gatei W (2021). Multisectoral cost analysis of a human and livestock anthrax outbreak in Songwe Region, Tanzania (December 2018–January 2019), using a novel Outbreak Costing Tool. One Health.

[121] Lewis N, Dorjee S, Dube C, VanLeeuwen J, Sanchez J (2017). Assessment of Effectiveness of Control Strategies against Simulated Outbreaks of Highly Pathogenic Avian Influenza in Ontario, Canada. Transbound Emerg Dis.

[122] Anderson A, Shwiff S, Gebhardt K, Ramírez AJ, Shwiff S, Kohler D, Lecuona L (2014). Economic evaluation of Vampire Bat (*Desmodus rotundus)* rabies Prevention in Mexico. Transbound Emerg Dis.

[123] Walker PGT, Cauchemez S, Metras R, Dung DH, Pfeiffer D, Ghani AC (2010). A bayesian Approach to quantifying the effects of Mass Poultry Vaccination upon the spatial and temporal dynamics of H5N1 in Northern Vietnam. PLoS Comput Biol.

[124] PREDICT Project. In: PREDICT Project. https://p2.predict.global. Accessed 9 Sep 2022.

[125] Loh EH, Zambrana-Torrelio C, Olival KJ, Bogich TL, Johnson CK, Mazet JAK, Karesh W, Daszak P (2015). Targeting transmission pathways for emerging zoonotic Disease Surveillance and Control. Vector-Borne and Zoonotic Diseases.

[126] Bordier M, Uea-Anuwong T, Binot A, Hendrikx P, Goutard FL (2020). Characteristics of one health surveillance systems: a systematic literature review. Prev Vet Med.

[127] Worsley-Tonks KEL, Bender JB, Deem SL (2022). Strengthening global health security by improving Disease surveillance in remote rural areas of low-income and middle-income countries. The Lancet Global Health.

[128] Reaser JK, Witt A, Tabor GM, Hudson PJ, Plowright RK (2021). Ecological countermeasures for preventing zoonotic Disease outbreaks: when ecological restoration is a human health imperative. Restor Ecol.

[129] Chen HL, Lewison RL, An L, Tsai YH, Stow D, Shi L, Yang S (2020). Assessing the effects of payments for ecosystem services programs on forest structure and species biodiversity. Biodivers Conserv.

[130] Chen Y, Marino J, Tao Q, Sullivan CD, Shi K, Macdonald DW (2016). Predicting hotspots of human-elephant conflict to inform mitigation strategies in Xishuangbanna, Southwest China. PLoS ONE.

[131] Whitfort A (2021). COVID-19 and Wildlife Farming in China: legislating to Protect Wild Animal Health and Welfare in the wake of a global pandemic. J Environ Law.

