# Supplementary material for: Policies to prevent zoonotic spillover: a systematic scoping review of evaluative evidence
Source: Global Health. 2023 Nov 8;19:82. doi: 10.1186/s12992-023-00986-x (PMC10634115; doi:10.1186/s12992-023-00986-x)
Supplement: Supplementary file 1 — Supplementary Material 1 [file 12992_2023_986_MOESM1_ESM.pdf]

Supplementary File 5

Results for quality assessment using tool informed by Dixon-Woods et al.

Author	Year	Fatally flawed? (yes/no - based on criteria assessment and relevance)	Clarity of aims and objectives (High/med/low)	Appropriateness of research design (High/med/low)	Clarity of research process (High/med/low)	Appropriateness and clarity of analysis (High/med/low)	Sufficiency of data to support interpretations and conclusions (High/med/low)	Relevance to the research question (high, medium, low)
Abbas	2014	No	Medium	Medium	High	Medium	High	High
Kung	2013	No	Medium	High	Medium	High	High	Medium
Häsler	2012	No	Medium	High	High	High	High	Medium
Horigan	2019	No	High	High	Medium	High	High	High
Ivančo Naletoski	2010	No	High	HIgh	High	Medium	High	High
R. Vivancos	2008	No	High	High	High	High	High	High
Catherine Brinkley	2018	No	High	High	High	High	High	High
Lauterbach	2020	No	High	High	High	High	High	High
Tustin	2011	No	High	High	Medium	Medium	High	High
Swayne	2011	No	Medium	High	High	High	High	High
Turkson	2016	No	Medium	High	Medium	Medium	High	Medium
Guerrier	2012	No	High	High	High	High	High	High
Akunzule	2019	No	Medium	Medium	Medium	Medium	High	High
Brooks-Moizer	2009	No	High	High	High	High	High	Medium
Knight-Jones	2011	No	High	Medium	High	High	High	High
Lin	2017	No	High	High	High	High	High	Medium
De Serres	2009	No	High	High	High	High	High	High
Shwiff	2009	No	High	High	High	High	High	High
Massey	2011	No	High	High	Medium	High	High	High

Huang	2017	No	Medium	High	High	High	High	Medium
Hunter	2014	No	Medium	Medium	High	High	High	Medium
Karki	2015	No	High	High	Medium	High	High	High
Kangas	2007	No	HIgh	High	Medium	High	High	High
Stewart	2017	No	High	High	High	High	High	High
Manyweathers	2017	No	High	High	High	High	High	High
Bonwitt	2018	No	High	High	High	High	High	Medium
Karabozhilova	2012	No	High	Medium	Medium	High	Medium	High
Sanchez	2020	No	High	High	High	High	High	High
Yee	2008	No	High	High	Medium	High	High	High
Huot	2008	No	High	High	Medium	High	High	High
Manyweathers	2017	No	High	High	High	High	High	High
Davies	2019	No	High	High	High	High	High	High
Gravier	2009	No	High	High	High	High	High	High
Thomas	2013	No	High	High	Medium	High	High	High
Gordon	2005	No	High	High	High	High	High	High
Mendez	2014	No	High	High	High	High	Medium	High
Okello	2018	No	High	Medium	High	High	Low	Medium
Lu	2009	No	High	Medium	High	High	High	High
Rasouli	2009	No	High	High	High	High	High	High
Wilson	2001	No	High	High	Low	Medium	High	High
