# Supplementary material for: Policies to prevent zoonotic spillover: a systematic scoping review of evaluative evidence
Source: Global Health. 2023 Nov 8;19:82. doi: 10.1186/s12992-023-00986-x (PMC10634115; doi:10.1186/s12992-023-00986-x)
Supplement: Supplementary file 2 — Supplementary Material 2 [file 12992_2023_986_MOESM2_ESM.pdf]

Supplementary File 4

Visualisation of study quality assessment using ROBINS-I

Risk of bias domains

Study	D1	D2	D3	D4	D5	D6	D7	Overall
1	X	-	+	+	+	+	+	X
2	X	-	+	+	+	+	+	X
3	-	+	+	+	+	+	+	-
4	-	+	+	+	+	+	+	-
5	-	+	+	+	+	+	+	-
6	-	-	+	+	+	+	+	-
7	-	+	+	+	+	+	+	-
8	-	+	+	+	+	+	+	-
9	-	+	+	+	+	+	+	-
10	-	+	+	+	+	+	+	-
11	-	+	+	+	+	+	+	-
12	-	+	+	+	!	+	+	!
13	-	+	+	+	+	+	+	-
14	-	+	+	+	+	-	+	-
15	X	+	+	+	+	+	+	X
16	-	+	+	+	+	+	+	-
17	-	+	+	+	+	+	+	-
18	-	+	+	+	+	+	+	-
19	-	+	+	+	+	+	+	-
20	-	+	+	+	+	+	+	-
21	-	+	+	+	+	+	+	-
22	-	+	+	+	+	+	+	-
23	-	+	+	+	+	+	+	-
24	-	+	+	+	+	+	+	-
25	-	+	+	+	+	+	+	-

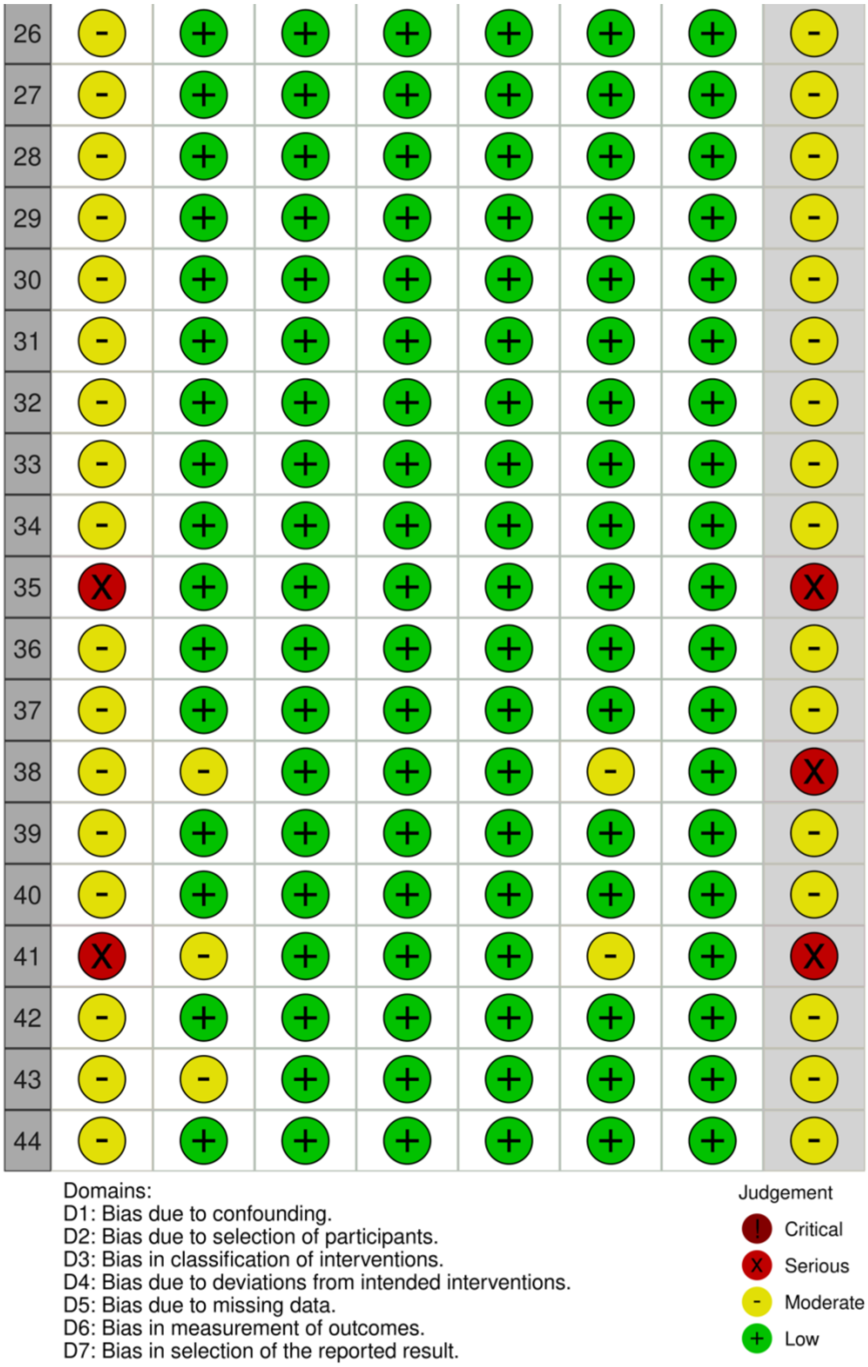

Visualisation generated using the *robvis* R package and Shiny web app:

McGuinness, LA, Higgins, JPT. Risk-of-bias VISualization (robvis): An R package and Shiny web app for visualizing risk-of-bias assessments. Res Syn Meth. 2020; 1- 7. https://doi.org/10.1002/jrsm.1411

Key to study numbers in ROBINS-I visualisation

Study number	Study title
1	Closure of live bird markets leads to the spread of H7N9 influenza in China
2	Shifting brucellosis risk in livestock coincides with spreading seroprevalence in elk
3	Impact of the implementation of rest days in live bird markets on the dynamics of H5N1 highly pathogenic avian influenza
4	The Impact of a Monthly Rest Day on Avian Influenza Virus Isolation Rates in Retail Live Poultry Markets in Hong Kong
5	The persistence of multiple strains of avian influenza in live bird markets
6	Protection of wetlands as a strategy for reducing the spread of avian influenza from migratory waterfowl
7	Effect of closure of live poultry markets on poultry-to-person transmission of avian influenza A H7N9 virus: an ecological study
8	A Bayesian Approach to Quantifying the Effects of Mass Poultry Vaccination upon the Spatial and Temporal Dynamics of H5N1 in Northern Vietnam
9	A little goes a long way: Weak vaccine transmission facilitates oral vaccination campaigns against zoonotic pathogens
10	A Moderateeling study of human infections with avian influenza A H7N9 virus in mainland China
11	A Network Control Theory Approach to Moderateeling and Optimal Control of Zoonoses: Case Study of Brucellosis Transmission in Sub-Saharan Africa
12	A retrospective study of anthrax on the Ghaap Plateau, Northern Cape province of South Africa, with special reference to the 2007–2008 outbreaks
13	An evaluation of the efficiency of rabies control strategies in fox (Vulpes Tulpes) populations using a computer simulation program
14	Application of a healthy food markets guide to two Indonesian markets to reduce transmission of “avian flu”
15	Assessing the rabies control and surveillance systems in Brazil: An experience of measures toward bats after the halt of massive vaccination of dogs and cats in Campinas, Sao Paulo
16	Assessment and simulation of the implementation of brucellosis control programme in an endemic area of the Middle East
17	Evaluation of the Efficacy of Intervention Measures and Vaccination for the Control of LPAI Epidemics in Verona Province (Veneto, Italy)
18	Public Health Benefits from Livestock Rift Valley Fever Control: A Simulation of Two Epidemics in Kenya
19	The European trade ban on wild birds reduced invasion risks Laura
20	Effect of Live Poultry Market Closure on Avian Influenza A (H7N9) Virus Activity in Guangzhou, China, 2014
21	A Simulation-Based Evaluation of Premovement Active Surveillance Protocol Options for the Managed Movement of Turkeys to Slaughter During an Outbreak of Highly Pathogenic Avian Influenza in the United States
22	Assessment of Effectiveness of Control Strategies Against Simulated Outbreaks of Highly Pathogenic Avian Influenza in Ontario, Canada

23 Economic Evaluation of Vampire Bat (DesModerateus rotundus) Rabies Prevention in Mexico

24 Highly pathogenic avian influenza H5N8 in south-west France 2016–2017: A Moderateeling study of control strategies

25 Poultry Market Closures and Human Infection with Influenza A(H7N9) Virus, China, 2013–14

26 Environmental Sampling for Avian Influenza A (H7N9) in Live-Poultry Markets in Guangdong, China

27 Metapopulation dynamics of rabies and the efficacy of vaccination

28 Transmission potential of influenza A/H7N9, February to May 2013, China

29 Controlling highly pathogenic avian influenza outbreaks: An epidemiological and economic Moderateel analysis

30 Moderateelling the effectiveness and risks of vaccination strategies to control classical swine fever epidemics

31 Moderateelling influenza A H5N1 vaccination strategy scenarios in the household poultry sector in Egypt

32 A mathematical Moderateel for the control of diseases in wildlife populations: culling, vaccination and fertility control

33 Contact reduction from live-poultry market closures limit the epidemic of human infection with H7N9

34 Different intervention strategies toward live poultry markets against avian influenza

35 Effects of closures of live poultry markets on poultry-to-person transmission of avian influenza A H7N9; an ecological study

36 Effect of intervention on the control of Highly Pathogenic Avian Influenza in Nigeria

37 Effectiveness of Live Poultry Market Interventions on Human Infection and Avian Influenza A (H7N9) Virus, China

38 Rift Valley Fever Virus in Egyptian Cattle and their Prevention

39 Quantitative Risk Assessment of the Introduction of Rabies into Japan Through Illegal Landing of Dogs from Russian Fishing Boats in the Ports of Hokkaido, Japan

40 Managing the Risk of Wildlife Disease Introduction: Pathway Level Biosecurtiy for Preventing the Introduction of Alien Ranaviruses

41 Live Poultry Market Closures and Avian Influenza A (H7N9) Infection in Cities in China 2013-2017

42 Interventions for Avian Influenza A (H5N1) Risk Management in Live Bird Market Networks

43 Effect of closure of live poultry marketes in China on prevention and control of human infection with H7N9 avian influenza: A case study of four cities in Jiangsu Province

44 Assessing reappearance factors of H7N9 avian influenza in China
