# Supplementary material for: Policies to prevent zoonotic spillover: a systematic scoping review of evaluative evidence
Source: Global Health. 2023 Nov 8;19:82. doi: 10.1186/s12992-023-00986-x (PMC10634115; doi:10.1186/s12992-023-00986-x)
Supplement: Supplementary file 3 — Supplementary Material 3 [file 12992_2023_986_MOESM3_ESM.pdf]

Supplementary File 3

Characteristics of included evaluations

First author	Year	Country	Disease(s)	Intervention sector	Implementation date	Policy type	Spillover pathway	Policy level	Governance sector	Study design	Key variables or concepts (Policy impact: direction of impact on outcome (+/-/=))
Li (1)	2018	China	H7N9 (Avian influenza)	Retail	Various local closures March 2013-March 2014	Market closure	Human exposure	Local	Health	Natural experiment	Human cases near closed markets (-); transportation of animals (+); human cases near other markets (+); price of broiler chickens near closed markets (-)
Abbas (2)	2014	Djibouti	BSE; Rift valley fever; brucellosis; contagious bovine pleuropneumonia	Trade	2007/2008 - following 2001/2002 outbreak, there was a ban on trade. However, trade ban was lifted in 2007 after efforts described in article were implemented.	Animal quarantine (testing, prophylaxis, treatment and culling)	Pathogen prevalence in domesticated animals	Regional	Animal health	Cross-sectional	Cases; culling rates; animals accepted/rejected for onward sale; treatments and prophylaxis; condition of animals on departure
Brennan (3)	2017	USA	Brucellosis	Agriculture and livestock	Various feeding grounds (1990-2014)	Supplemental feeding of wildlife	Wildlife distribution and density	Local	Agriculture; national parks and conservation	Case-control	Brucellosis-affected livestock herds near fed elk (-) vs unfed elk (+)
Fournié (4)	2011	Hong Kong	H5N1 (Avian influenza)	Retail	N/A	Rest days	Domesticated animal pathogen survival and spread	Local	N/A	Modelling	Flock reproduction number r, defined as the expected number of secondary cases (market or farm) per single infected case in a fully susceptible population (-)
Kung (5)	2003	Hong Kong	H9N2 (Avian influenza);	Retail	Jun-01	Rest days	Domesticated animal pathogen	Local	Not stated	Natural experiment	Prevalence of H9N2 in faecal samples from

First author	Year	Country	Disease(s)	Intervention sector	Implementation date	Policy type	Spillover pathway	Policy level	Governance sector	Study design	Key variables or concepts (Policy impact: direction of impact on outcome (+/-/=))
			Newcastle disease virus				survival and spread				LBMs, post vs. pre-rest days (-); prevalence of Newcastle disease virus in faecal samples from LBMs, post vs. pre-rest days (=)
Kung (6)	2013	Australia	Hendra	Agriculture and livestock	Not stated	Recommendations for livestock owners (stable horses overnight; place water away from trees; isolate sick horses and seek veterinary advice)	Domesticated animal exposure; pathogen prevalence in domesticated animals	Local	Not stated	Cross-sectional	Awareness of state information on Hendra virus and risks management strategies among horse owners; risk management practices among horse owners; perception of state advice among horse owners (e.g. advice is not in layman's terms, expensive to implement, focus should be on flying fox control)
Pinsent (7)	2017	China	Avian influenza	Retail	N/A	Market size	Domesticated animal distribution and density	Local	N/A	Modelling	Prevalence of two strains of avian influenza (-)
Wu (8)	2019	China	H5N1 (Avian influenza)	Conservation and national parks	Prior to data collection start	Habitat protection	Wildlife distribution and density	Global	Agriculture; national parks and conservation	Case-control	Risk of outbreak of H5N1 (-)
Yu (9)	2014	China	H7N9 (Avian influenza)	Retail	Apr-13	Market closure	Human exposure	Local	Health	Natural experiment	Poultry-to-person transmission of H7N9 (-)
Walker (10)	2010	Vietnam	H5N1 (Avian influenza)	Agriculture and livestock	2005	Mandatory livestock vaccination	Within-domesticated animal barriers	National	Animal health	Natural experiment	Transmission between farms (-); time taken to identify outbreaks (+)

First author	Year	Country	Disease(s)	Intervention sector	Implementation date	Policy type	Spillover pathway	Policy level	Governance sector	Study design	Key variables or concepts (Policy impact: direction of impact on outcome (+/-/=))
Basinski (11)	2019	USA	Rabies	Conservation and national parks	N/A	Transmissible vaccination using oral bait	Pathogen prevalence in wildlife	National	N/A	Modelling	Seroprevalence of rabies (+)
Liu (12)	2015	China	H7N9 (Avian influenza)	Agriculture and livestock	N/A	Screen and cull	Pathogen prevalence in domesticated animals	National	N/A	Modelling	New human H7N9 cases (-)
Roy (13)	2011	N/A	Brucellosis	Agriculture and livestock	N/A	Mandatory livestock vaccination	Within-domesticated animal barriers	N/A	N/A	Modelling	Basic reproductive ratio (-)
Roy (13)	2011	N/A	Brucellosis	Agriculture and livestock	N/A	Screen and cull	Pathogen prevalence in domesticated animals	N/A	N/A	Modelling	Basic reproductive ratio (-)
Häsler (14)	2012	Switzerland	Avian influenza	Agriculture and livestock; conservation and national parks	Sep-05	Multi-component strategy	Multiple points	National	Animal health	Cost-effectiveness analysis	Probability of primary and secondary HPAIV outbreaks in poultry with active surveillance in place relative to the situation with no active surveillance
Horigan (15)	2019	United Kingdom	Avian influenza	Agriculture and livestock	2005	Disinfection of livestock premises	Domesticated animal pathogen survival and spread	Regional	Not stated	Qualitative risk assessment	Probability of virus survival on different types of equipment in a depopulated contaminated poultry house before and after preliminary and secondary C&D procedures
Hassim (16)	2017	South Africa	Anthrax	Agriculture and livestock	2007-2008	Carcass containment (burning, disinfecting or putting in plastic bags)	Wildlife pathogen survival and spread	National	Agriculture, fisheries and forestry	Repeat cross-sectional pre-post quasi-experimental	Anthrax present at carcass disposal sites (-)

First author	Year	Country	Disease(s)	Intervention sector	Implementation date	Policy type	Spillover pathway	Policy level	Governance sector	Study design	Key variables or concepts (Policy impact: direction of impact on outcome (+/-/=))
Selhorst (17)	1999	N/A	Rabies	Conservation and national parks	N/A	Vaccination using oral bait	Pathogen prevalence in wildlife	N/A	N/A	Modelling	Immunisation rate (+)
Samaan (18)	2012	Indonesia	Avian influenza	Retail	2006	Information campaign	Domesticated animal pathogen survival and spread	Global	Health	Repeat cross-sectional pre-post non-experimental	Knowledge and practices to limit spread of avian influenza among stallholders (+)
De Lucca (19)	2013	Brazil	Rabies	Agriculture and livestock	2010	Targeted vaccination of domestic animals	Within-domesticated animal barriers	National	Animal health	Cross-sectional post-test only quasi-experimental	Probability of infectious contact between dogs and cats and rabies-positive bats in areas with targeted vaccination versus control areas (+)
Hegazy (20)	2009	Egypt	Brucellosis	Agriculture and livestock	Not stated	Screen and cull	Pathogen prevalence in domesticated animals	National	Animal health	Modelling	Prevalence of brucellosis (-)
Busani (21)	2007	Italy	Avian influenza	Agriculture and livestock	2000	Livestock vaccination	Within-domesticated animal barriers	Local	Animal health	Repeat cross-sectional pre-post quasi-experimental	Risk of infection (-)
Kimani (22)	2016	Kenya	Rift Valley Fever	Agriculture and livestock	N/A	Livestock surveillance + improved vaccination coverage	Pathogen prevalence in domesticated animals	National	N/A	Modelling	DALYs averted (-); cost-effectiveness for public health system (+)
Cardador (23)	2019	Spain and Portugal	Avian influenza	Trade	Oct, 2005	Wildlife trade ban	Human exposure	Regional	Not stated	Longitudinal interrupted time series non-experimental	(1) change in introduction numbers: numbers of new nonnative bird species annually recorded; (-) after the EU ban. (2) bird availability for sale in local pet markets; (=) after the ban.
Bonwitt (24)	2018	Sierra Leone	Ebola virus disease	Production and trade	Not stated	Wild meat ban (hunting, sale, and consumption)	Human exposure	National	Health	Qualitative semi-	Lived experiences and accounts of participant's

First author	Year	Country	Disease(s)	Intervention sector	Implementation date	Policy type	Spillover pathway	Policy level	Governance sector	Study design	Key variables or concepts (Policy impact: direction of impact on outcome (+/-/=))
										structured interviews	understanding and interpretation of public health messages related to EVD and wild animals, and how these understandings affected hunting, consumption, and trade of wild meat.
Yuan (25)	2015	China	Avian Influenza A(H7N9) Virus	Retail	2014	Market closure and Disinfection of livestock premises	Domesticated animal pathogen survival and spread	Local	Health	Repeat cross-sectional pre-post non-experimental	Detection rates for H7N9 and AIV during market closure (-) and after market re-opening (=)
Naletoski (26)	2010	Macedonia	Brucellosis	Agriculture and livestock	Mid 1970s	Screen and cull	Pathogen prevalence in domesticated animals	National	Animal health; Agriculture and environment	Descriptive cross-sectional study	Expected and actual number of sampled (tested) sheep and goats; absolute number of positive animals; number of infected vs non-infected holdings; prevalence of brucellosis; disease prevalence in the investigated villages; and removal rate of positive animals between 2000 and 2006.
Vivancos (27)	2008	UK	Avian influenza	Public health	2007	Vaccination of poultry workers	Within-human barriers	National	Health	Rapid cross-sectional evaluation	Delivery, uptake and costs of seasonal influenza vaccine among poultry workers; problems

First author	Year	Country	Disease(s)	Intervention sector	Implementation date	Policy type	Spillover pathway	Policy level	Governance sector	Study design	Key variables or concepts (Policy impact: direction of impact on outcome (+/-/=))
											encountered; ways of improving delivery
Brinkley (28)	2018	USA	Avian influenza	Agriculture and livestock	1905-2016	Backyard poultry ordinances (e.g. vaccination, veterinary care, number of animals allowed)	Multiple points	Local	Animal health; Agriculture	Comparative analysis	Poultry ordinances passed in Colorado; guidelines for the health and welfare of backyard poultry; shelter and welfare agency intake
Smith (29)	2001	England	Rabies and Bovine Tuberculosis	Conservation and health	N/A	Culling; sterilisation; vaccination	Wildlife density & distribution; pathogen prevalence in wildlife	N/A	N/A	Modelling	Disease eradication (+)
Weaver (30)	2016	USA	Avian influenza	Agriculture and livestock	N/A	Screen and remove	Pathogen prevalence in domesticated animals	National	N/A	Modelling	Likelihood of detecting HPAI virus where present (+)
Lauterbach (31)	2020	USA	Influenza A virus and antimicrobial resistant coliform bacteria	Agriculture and livestock	Not stated	Recommendations for agricultural fairs (Hand sanitation stations, signage on risk, signage on washing procedure)	Human exposure	Local	Animal health; human health	Cross-sectional	Antimicrobial resistant coliform bacteria (+); influenza A virus was detection (-); utilization of educational signage (=);presence of hand sanitation stations at fairs (+) ; proportion of fairgoers who use hand sanitation stations (-)
Tustin (32)	2011	Iceland	Campylobacteriosis	Agriculture and livestock	1995 to 2007	Multi-component strategy (Biosecurity measures and producer education,	Domesticated animal pathogen survival and spread;	National	Animal health; food safety		On-farm surveillance of Campylobacter; producer and consumer education;

First author	Year	Country	Disease(s)	Intervention sector	Implementation date	Policy type	Spillover pathway	Policy level	Governance sector	Study design	Key variables or concepts (Policy impact: direction of impact on outcome (+/-/=))
						campylobacter surveillance in poultry, interventions in poultry processing)	human exposure				enhanced biosecurity measures; changes in poultry processing; leak-proof packaging policy; freezing policy for products from Campylobacter-positive poultry flocks; multidisciplinary response committee
Lewis (33)	2015	Canada	Avian influenza (HPAI)	Agriculture and livestock	N/A	Multi-component strategy (Screen and cull; movement restrictions; biosecurity measures)	Multiple points (Pathogen prevalence in domesticated animals; domesticated animal pathogen survival and spread)	Local	N/A	Modelling	Number of infected flocks (--)
Lewis (33)	2015	Canada	Avian influenza (HPAI)	Agriculture and livestock	N/A	Multi-component strategy (Screen and cull; ring cull; movement restrictions; biosecurity measures)	Multiple points (Pathogen prevalence in domesticated animals; domesticated animal pathogen survival and spread)	Local	N/A	Modelling	Number of infected flocks (-)
Swayne (34)	2011	Multiple Countries	Avian influenza	Agriculture and livestock	Various dates between 2002 and 2010	Livestock vaccination, including as part of a multi-component strategy	Within-domesticated animal barriers	National	Not stated	Cross-sectional	Avian influenza (AI) control strategies; general AI vaccine use policy; AI vaccine bank; vaccine usage;

First author	Year	Country	Disease(s)	Intervention sector	Implementation date	Policy type	Spillover pathway	Policy level	Governance sector	Study design	Key variables or concepts (Policy impact: direction of impact on outcome (+/-/=))
											vaccination strategy; vaccine licensing; national poultry and egg production; national poultry density; HPAI and LPNAI outbreaks and the species affected; AI vaccine coverage (%)
Turkson (35)	2016	Ghana	Avian influenza	Agriculture and livestock	2007	Multiple strategies (biosecurity; reporting; culling with compensation; movement control)	Multiple points	National	Animal health	Cross-sectional (closed and open-ended questions)	Practices, capacity and incentives in terms of implementing peacetime preventive mitigation measures (biosecurity and reporting) among poultry supply chain actors and outbreak containment measures (culling with compensation and movement control) among poultry supply chain actors
Guerrier (36)	2012	Wallis and Futuna (French Polynesia)	Brucellosis	Agriculture and livestock	2004 and 2008	Information campaign	Human exposure	Local	Animal health; public health	Cross-sectional; qualitative semi-structured interviews	Level of knowledge of brucellosis; local hygiene standards and veterinary access; local understanding of pig-associated illness; adequacy and cultural appropriateness

First author	Year	Country	Disease(s)	Intervention sector	Implementation date	Policy type	Spillover pathway	Policy level	Governance sector	Study design	Key variables or concepts (Policy impact: direction of impact on outcome (+/-/=))
											of public health measures
Anderson (37)	2012	Mexico	Rabies	Agriculture and livestock	1997 and 2006	Livestock vaccination	Within-domesticated animal barriers	National	N/A	Modelling	Benefit-cost ratio (+)
Anderson (37)	2012	Mexico	Rabies	Conservation	1997 and 2006	Wildlife cull	Wildlife distribution and density	National	N/A	Modelling	Benefit-cost ratio (-)
Akunzule (38)	2019	Ghana	Avian Influenza (HPAI)	Agriculture and livestock	2006-2007	Multiple strategies (destruction of infected eggs; culling; decontamination; movement control)	Multiple points	National	Animal health	Cross-sectional; qualitative semi-structured interviews and focus groups	Direct costs associated with HPAI prevention and control measures; loss in expected output due to the presence of HPAI; losses due to fall in domestic prices and demand; cost of HPAI prevention measures (such as cost of vaccine to control the disease, cost of disinfectant, foot bath, and other related costs to improve biosecurity); Benefits - the summation of the avoided losses of the expected output and the decrease in the cost of prevention
Andronico (39)	2019	France	Avian influenza (HPAI)	Agriculture and livestock	2016–2017	Screen and cull	Pathogen prevalence in domesticated animals	National	N/A	Modelling	Number of infected premises (-); number of animals culled (-)
Wu (40)	2014	China	Influenza A(H7N9)	Retail	Various local closures in 2014	Market closure	Human exposure	National	Not stated	Natural experiment (non-	Human risk for H7N9 infection (-)

First author	Year	Country	Disease(s)	Intervention sector	Implementation date	Policy type	Spillover pathway	Policy level	Governance sector	Study design	Key variables or concepts (Policy impact: direction of impact on outcome (+/-/=))
										experimental repeat cross-sectional; number of time points not specified)	
Kang (41)	2015	China	Avian Influenza A (H7N9)	Retail	March 2013 – June 2014	Market closure	Human exposure	Local	Health	Repeat cross-sectional pre-post non-experimental	Proportion of H7N9-positive environmental samples
Beyer (42)	2010	Tanzania	Rabies	Public Health	Multiple vaccination campaigns 2000-2006	Vaccination in domesticated animals	Within-domesticated animal barriers	Not stated	Public Health; Livestock and fisheries	Modelling	Rabies occurrence as indicated by patients reported with bites from suspected rabid dogs (-)
Chowell (43)	2013	China	Influenza A (H7N9)	Retail	Apr-13	Market closure	Human exposure	Not stated	Public Health	Repeat cross-sectional interrupted time series non-experimental	Reproduction number (R) of A/H7N9 (-)
Backer (44)	2015	Netherlands	Avian influenza (HPAI)	Agriculture and livestock	N/A	Multi-component strategy	Multiple points	Regional	N/A	Modelling	Relative to pre-emptive cull and emergency vaccination: Epidemic duration (+); number of culled farms (=)
Backer (44)	2015	Netherlands	Avian influenza (HPAI)	Agriculture and livestock	N/A	Pre-emptive cull	Pathogen prevalence in domesticated animals	Regional	N/A	Modelling	Relative to multi-component strategy and emergency vaccination: Epidemic duration (-); number of culled farms (+)
Backer (44)	2015	Netherlands	Avian influenza (HPAI)	Agriculture and livestock	N/A	Emergency vaccination	Within-domesticated animal barriers	Regional	N/A	Modelling	Relative to multi-component strategy and pre-emptive cull: number of culled farms (-); Epidemic duration relative to multi-

First author	Year	Country	Disease(s)	Intervention sector	Implementation date	Policy type	Spillover pathway	Policy level	Governance sector	Study design	Key variables or concepts (Policy impact: direction of impact on outcome (+/-/=))
											component strategy (-) and pre-emptive cull (+)
Brooks-Moizer (45)	2009	Vietnam	Avian Influenza H5N1	Trade	2005	Trade ban	Human exposure	National	Animal Health	Cross-sectional; qualitative semi-structured interviews	Volume of wild bird trade; number of species on sale; vendor awareness and understanding of legislation
Backer (46)	2008	Netherlands	Swine fever	Agriculture and livestock	N/A	Multi-component strategy	Multiple points	Regional	N/A	Modelling	Relative to pre-emptive culling and vaccination: Epidemic duration (+); pre-emptively culled farms (-)
Backer (46)	2008	Netherlands	Swine fever	Agriculture and livestock	N/A	Pre-emptive cull	Pathogen prevalence in domesticated animals	Regional	N/A	Modelling	Relative to multi-component strategy and livestock vaccination: Epidemic duration (-, except for 5km ring vaccination); number of culled farms (+)
Backer (46)	2008	Netherlands	Swine fever	Agriculture and livestock	N/A	Livestock vaccination	Within-domesticated animal barriers	Regional	N/A	Modelling	Relative to multi-component strategy and pre-emptive cull: Epidemic duration (= for smaller vaccination radius (lower than multi-component and higher than culling); - for larger vaccination radius); number of culled farms relative to multi-component strategy (+) and

First author	Year	Country	Disease(s)	Intervention sector	Implementation date	Policy type	Spillover pathway	Policy level	Governance sector	Study design	Key variables or concepts (Policy impact: direction of impact on outcome (+/-/=))
											pre-emptive culling (-)
El Masry (47)	2013	Egypt	Influenza A H5N1	Agriculture and livestock	N/A	Livestock vaccination	Within-domesticated animal barriers	National	N/A	Modelling	Cumulative annual flock immunity (=)
Knight-Jones (48)	2011	United Kingdom	Avian influenza (HPAI)	Agriculture and livestock	2006	Separation of wildlife and livestock	Domesticated animal exposure	National	Animal Health	Cross-sectional	Implementation of recommendations (separation of wild and domestic birds) by poulty farmers; cost and welfare issues relating to implementing the recommendations
Xing (49)	2017	China	H7N9 Avian Ifluenza	Retail	N/A	Market closure	Human exposure	N/A	N/A	Modelling	Theoretic number of human cases: no market closure (+); markets closed (short term) (- then +); markets closed (no reopening (-))
Lin (50)	2017	China	H7N9	Agriculture and livestock; retail	Late 2014	Central slaughtering	Human exposure	Local	Not stated	Cross-sectional	Demographics; knowledge and perception of avian flu; perception of the CSLPP; influential on attitudes towards the CSLPP
De Serres (51)	2009	Canada	Rabies	Public Health	1998, update in 2006	Post-exposure prophylaxis	Within-human barriers	National	Public Health	Risk-benefit analysis	Proportion of population exposed annually; Proportion of RPEP-eligble person reached; Incidence of preventable disease; NNT per case prevented; human and

First author	Year	Country	Disease(s)	Intervention sector	Implementation date	Policy type	Spillover pathway	Policy level	Governance sector	Study design	Key variables or concepts (Policy impact: direction of impact on outcome (+/-/=))
											material resources required per case prevented
Shwiff (52)	2009	United States	Rabies	Conservation and National Parks	N/A	Wildlife vaccination	Pathogen prevalence in wildlife	Local	N/A	Cost-benefit analysis	Costs: area of bait application; bait price; bait density; campaign frequency; mode of bait application; level of prevention; contingency expenditure. Benefits (cost savings): direct - copayment of PEP; indirect - costs of human exposure and animal testing
Massey (52)	2011	Australia	Swine brucellosis	Hunting	2010	Recommendations for hygiene practices (recommended risk reduction strategies for feral pig hunters harvesting wild boar meat to prevent swine brucellosis, e.g. cover cuts, wear gloves, wash hands)	Human exposure	Local	Public Health (Food safety)	Qualitative interviews and focus groups	Appropriateness of current swin brucellosis risk reduction strategies; potential alternative strategies; apropriateness of information dissemination; potential dissemination ideas
Huang (53)	2017	China	HPAI	Livestock and agriculture	Not stated	Safe disposal of infected livestock	Domesticated animal pathogen survival and spread	National	Animal health	Cross-sectional	Acceptable compensation levels; location; gender; age; education; farm size; net income; % net income from farm; contact with

First author	Year	Country	Disease(s)	Intervention sector	Implementation date	Policy type	Spillover pathway	Policy level	Governance sector	Study design	Key variables or concepts (Policy impact: direction of impact on outcome (+/-/=))
											epidemic prevention staff
Hunter (54)	2014	Indonesia	HPAI	Agriculture and livestock	2010	Multi-component strategy	Multiple points	National	Not stated	Cross-sectional survey; focus groups; interviews	Demographic data; poultry flock management; management of sick/dead birds; HPAI knowledge and perceptions; HPAI occurrences; HPAI activities (information sources and information flow)
Teng (55)	2018	China	Avian influenza (H7N9)	Retail	2013	Market closures	Human exposure	Local	Public Health	Modelling	Number of confirmed cases after live poultry market closures (-); number of human cases in latent population after market closures (-)
Karki (56)	2015	Nepal	Avian influenza H5N1	Animal Health	2007	Multi-component strategy	Multiple points	National	Not stated	Cost-benefit analysis	Cost under control program (surviellance; stamping out operations and compensation; trainging, communication and information) losses under current control progam (losses due to morality cause by HPAI H5N1; losses due to culling of poultry; losses due to production ban period; losesses due to market reaction); absence of

First author	Year	Country	Disease(s)	Intervention sector	Implementation date	Policy type	Spillover pathway	Policy level	Governance sector	Study design	Key variables or concepts (Policy impact: direction of impact on outcome (+/-/=))
											control measures (losses due to HPAI mortality; losses due to market reaction); vaccine programme costs (cost of vaccine; cost of administering vaccine; cost of surveillance; cost of stamping-out operations and compensation; cost of training, communication and information dissemination); losses under vaccination program (losses due to HPAI mortality; losses due to culling)
Kangas (57)	2007	Finland	Salmonellosis	Agriculture and livestock	1995	Multi-component strategy	Multiple points	National	Not stated	Cost-benefit analysis	Control costs; public health losses (Official salmonella control costs; additional control of primary and secondary production; market disturbances; feed control; additional)not FSCP) control costs to society; public health losses; losses due to premature death)

First author	Year	Country	Disease(s)	Intervention sector	Implementation date	Policy type	Spillover pathway	Policy level	Governance sector	Study design	Key variables or concepts (Policy impact: direction of impact on outcome (+/-/=))
Zhu (58)	2020	China	Avian influenza (H7N9)	Retail	N/A	Market closures	Human exposure	Local	N/A	Modelling	Weekly number of human cases (-); cumulative number of human cases (-)
Zhu (58)	2020	China	Avian influenza (H7N9)	Retail	N/A	Multi-component market management policy (disinfection, cleaning, monthly rest day, zero poultry overnight)	Domesticated animal pathogen survival and spread	Local	N/A	Modelling	Weekly number of human cases (-); cumulative number of human cases (-)
Stewart (59)	2017	United States	Influenza A (H3N2v)	Livestock and agriculture	2013, update November 2016	Recommendations for agricultural fairs (Hand sanitation stations, signage on risk, signage on washing procedure)	Human exposure	National	Animal health; public health	Cross-sectional	Demographic data (gender; age; number of household members attended fairs (with or without outbreak), uptake of season influenza vaccine); behaviours (practices); attitudes; support; knowledge
Oladokun (60)	2012	Nigeria	HPAI influenza A (H5N1)	Trade; Agriculture and livestock; Animal health	2006	Multi-component strategy (Total ban on import of poultry and poultry products, national borders were monitored, surveillance of live bird markets and control posts were intensified, proper disinfection of equipment in laboratory settings, disposal of carcasses)	Multiple points	National	Agriculture; Animal Health	Repeat cross-sectional interrupted time series non-experimental	Human cases of HPAI (-)

First author	Year	Country	Disease(s)	Intervention sector	Implementation date	Policy type	Spillover pathway	Policy level	Governance sector	Study design	Key variables or concepts (Policy impact: direction of impact on outcome (+/-/=))
Karabozhilova (61)	2012	UK	Avian disease	Agriculture and livestock	2001	Ban on feeding catering waste to livestock	Domesticated animal exposure	National	Agriculture	Cross-sectional	Compliance with the ban on feeding catering waste
Manyweathers (62)	2017	Australia	Hendra virus	Livestock and Agriculture	Not stated	Recommended livestock vaccination	Within-domesticated animal barriers	Local	Agriculture	Cross-sectional survey (closed and open-ended questions)	Attitudes to the vaccine; assesment of risk; attitudes to authorities; factors that influence the potential uptake of vaccination
Sanchez (63)	2020	United States	Rabies and Canine distemper virus	Conservation	2001 (rabies) 2000 (CDV)	Vaccination and surveillance	Pathogen prevalence in wildlife	Local	Conservation	Modelling	Number of sentinels monitored and monitoring frequency (+), earlier epidemic detection, infected foxes on day of detection (-)
Yee (64)	2008	United States	Avian influenza	Retail; Livestock and Agriculture	2005	LBM biosecurity measures (Direct delivery of birds to farms, carcass disposal through rendering services, control program)	Multiple points	Local	Food and Agriculture, Animal Health	Cross-sectional	Compliance with biosecurity measures
Huot (65)	2008	Canada	Rabies	Public Health	2002	Human vaccination (post-exposure prophylaxis)	Within-human barriers	National	Public Health	Cross-sectional	Type of exposure; bat virological analysis; cost of investigation, virological analysis and RPEP
Manyweathers (66)	2017	Australia	Hendra virus	Livestock and Agriculture	2015	Multi-component strategy (Covering horses' food and water containers, keeping horses off pasture when flying foxes are active, keeping	Domesticated animal exposure	Local	Livestock and Agriculture	Cross-sectional	Uptake of recommended risk mitigation strategies, factors influencing uptake; coping factors; affective factors; attitude

First author	Year	Country	Disease(s)	Intervention sector	Implementation date	Policy type	Spillover pathway	Policy level	Governance sector	Study design	Key variables or concepts (Policy impact: direction of impact on outcome (+/-/=))
						horses away from flowering trees.)					to hendra virsus risk
Wang (67)	2020	China	H7N9 Avian Ifluenza	Retail	Multiple local closures2013-2018	Live poultry market closure	Human exposure	Local	Not stated	Repeat cross-sectional interrupted time series non-experimental	Daily incidence rates (-)
Mroz (68)	2017	Egypt	Rift Valley Fever	Livestock and Agriculture	1980	Livestock vaccination	Within-domesticated animal barriers	National		Serological cross-sectional study	RFVF anti-body prevalence
Davis (69)	2019	United States	Rabies	Conservation and Wildlife	1995	Wildlife vaccination (oral rabies vaccination)	Pathogen prevalence in wildlife	National	Agriculture; wildlife; animal health	Longitudinal interrupted time series non-experimental	Rabies occupancy (-), duration of baiting and bait density influence seroprevalence in raccoons
Kwan (70)	2016	Japan	Rabies	Trade	Nov-04	Multi-component strategy (Identification with microchip, two-time rabies vaccination, antibody level titration and 180-day waiting period in country of origin.)	Pathogen prevalence in domestic animals	National	Foreign affairs; Agriculture; Public Health	Modelling	Risk of rabies introduction (-); risk of rabies introduction as a result of one Russian fishing boat arriving at the port of Hokkaido (-); theoretical number of Russian boat arrivals required to result in one rabies case (-); Annual probability that at least one rabies case is introduced (-); number of years required to elapse for the introduction of one rabies case (+)

First author	Year	Country	Disease(s)	Intervention sector	Implementation date	Policy type	Spillover pathway	Policy level	Governance sector	Study design	Key variables or concepts (Policy impact: direction of impact on outcome (+/-/=))
Garcia-Diaz (71)	2016	Australia	Ranaviruses	Trade	Not stated	Border and post-border biosurveillance and biosecurity	Pathogen prevalence in wildlife	National	Not stated	Modelling	Absolute risk of introduction of alien ranavirus (-); absolute risk of infected alien amphibian stowaways (-)
Graiver (72)	2009	United States	Avian Influenza (H6N2)	Livestock and Agriculture	2006	Disposal regulations for infected carcasses	Domesticated animal pathogen survival and spread	National	Environment	Cross-sectional	Avian influenza virus survival
Chen (73)	2020	China	Avian Influenza A (H7N9)	Retail	Multiple closures 2013-2017	Live poultry market closure	Human exposure	Local	Public health	Repeat cross-sectional interrupted time series non-experimental	Relative risk of H7N9 infection (-)
Fournié (74)	2013	Vietnam	HPAIV H5N1	Trade	N/A	Disinfection	Domesticated animal pathogen survival and spread	N/A	N/A	Modelling (Social network analysis)	Median epidemic size (-)
Ma (75)	2019	China	Avian Influenza A (H7N9)	Retail	Multiple closures 2013-2017	Live poultry market closures	Human exposure	Local		Case-control	Incidence of H7N9 cases (-)
Thomas (76)	2013	Grenada	Rabies	Animal health	Not stated	Domestic animal vaccination	Within-domesticated animal barriers	National	Agriculture; Public Health	Cross-sectional	Behavioural beliefs, control beliefs, normative beliefs, risk perception
Gordon (77)	2005	United States	Rabies	Conservation	1997 in Ohio, 2001 extended to Pennsylvania and West Virginia	Wildlife vaccination (oral rabies vaccination)	Pathogen prevalence in wildlife	Local	Agriculture; wildlife; public health	Modelling	Costs; cost savings; estimate of advance (km/year)
Mendez (78)	2014	Australia	Hendra virus (HeV)	Veterinary Health	2010	Multi-component strategy (HeV exposure management by veterinarians (e.g., use of PPE))	Human exposure	Local	Animal health	Cross-sectional	Professional, veterinary practice, and HeV management experience; HeV management strategies; PPE usage

First author	Year	Country	Disease(s)	Intervention sector	Implementation date	Policy type	Spillover pathway	Policy level	Governance sector	Study design	Key variables or concepts (Policy impact: direction of impact on outcome (+/-/=))
Okello (79)	2018	Lao PDR	T. solium taeniasis-cysticercosis	Public health	Not stated	Mass drug administration in humans	Within-human barriers	National	Health and Agriculture	Modelling	Cost per DALY averted relative to treating pigs and humans (-)
Okello (79)	2018	Lao PDR	T. solium taeniasis-cysticercosis	Public health	Not stated	Mass drug administration in humans and livestock	Multiple points (within-human barriers, pathogen prevalence in domesticated animals)	National	Health and Agriculture	Modelling	Cost per DALY averted relative to treating humans only (+)
Lu (80)	2009	Saudi Arabia	H5N1 HPAI	Livestock and Agriculture	2007	Multi-component strategy (Live bird market closures, surveillance tests on poultry species, "stamping out", quarantine restrictions, training for veterinarians on biosecurity measures and sample collection, hygienic disposal of poultry carcasses and infectious waste)	Multiple points (Pathogen prevalence in domesticated animals; human exposure)	National	Not stated	Ecological study using multiple methods	Number of affected farms; location of outbreaks; implementation of control measures
Rasouli (81)	2009	Azerbaijan	Brucellosis	Livestock and Agriculture	Not stated	Livestock vaccination	Within-domesticated animal barriers	Local	Not stated	Modelling	Net profit (+), costs (-), cost-benefit ratio (+)
Wilson (82)	2001	United States	Rabies	Animal Health	1979, amended 1988	Vaccination in domesticated animals	Within-domesticated animal barriers	Local	Public health	Retrospective surveillance	Number of animals receiving PEP; PEP failure rate; characteristics of PEP failure cases (age, date of exposure, date of vaccination/PEP administration, date of rabies incident)

First author	Year	Country	Disease(s)	Intervention sector	Implementation date	Policy type	Spillover pathway	Policy level	Governance sector	Study design	Key variables or concepts (Policy impact: direction of impact on outcome (+/-/=))
Wang (83)	2018	China	H7N9 Avian Influenza	Retail	3 closure periods: 10-23 Feb 2014; 19-28 Feb 2015; 8-21 Jan 2017	Market closure	Human exposure	Local	Public Health	Natural experiment	Relative risk of H7N9 infection
Lyu (84)	2014	China	H7N9 Avian Influenza	Retail	Multiple staggered 3-month market closures from Jan 2014 onwards	Market closure and disinfection of livestock premises	Human exposure	Local	Public Health	Natural experiment	Number of human cases of H7N9 infection
Cuthbert (85)	2011	India	Multiple	Livestock and agriculture	2006 (withdrawal of permits to manufacture); 2008 (use of diclofenac an imprisonable offence)	Ban on veterinary drugs dangerous to wildlife	Wildlife distribution and density	National	Conservation; public health (drug policy)	Modelling	Prevalence of diclofenac in domestic ungulate carcasses (-); concentration of diclofenac in domestic ungulate carcasses (-); rate of decline of vulture populations (-)
Weaver (86)	2016	Japan	Rabies	Animal Health	1950; amended 1998	Mandatory dog registration and vaccination	Pathogen prevalence in domestic animals	National	Animal health; border control	Cross sectional; qualitative interviews and document analysis	Cases of rabies; implentation; roles and responsibilities
Weaver (86)	2016	Japan	BSE	Animal Health; border control	Ongoing review (2001; 2009)	Screen and cull	Pathogen prevalence in domesticated animals	National	Animal Health	Qualitative interviews and document analysis	Policy learning
Weaver (86)	2016	Japan	Multiple	Border control	Not stated	Quarantine and border security for livestock and animal food items	Pathogen prevalence in domesticated animals	National	Animal Health; Food safety	Document analysis	Resourcing; organisational structure and collaboration; implementation
Weaver (86)	2016	Japan	Multiple	Animal Health	Not stated	Annual biosecurity inspections of livestock premises	Pathogen prevalence in domesticated animals	National	Animal health	Document analysis	Funding adequacy; implementation

First author	Year	Country	Disease(s)	Intervention sector	Implementation date	Policy type	Spillover pathway	Policy level	Governance sector	Study design	Key variables or concepts (Policy impact: direction of impact on outcome (+/-/=))
Weaver (86)	2016	Japan	HPAI	Border control	Not stated	Import ban on livestock from areas with HPAI	Pathogen prevalence in domesticated animals	National	Animal health	Document analysis	Resourcing; organisational structure and collaboration; implementation
Amparo (87)	2018	Philippines	Rabies	Public health	2016	Post-exposure prophylaxis	Within-human barriers	National	Public Health	Repeat cross-sectional survey	Animal bite/scratch incidence; health-seeking behaviour; Out of pocket PEP expenses
Ferguson (88)	2015	Philippines	Rabies	Animal Health	2010-2012 (3 vaccination campaigns)	Domestic animal vaccination	Within-domesticated animal barriers	Global	Public Health	Modelling	Vaccination coverage (+); rabies elimination (-)
Shwiff (89)	2016	South Africa	Rabies	Animal Health; public health	2007	Domestic animal vaccination; post-exposure prophylaxis	Within-domesticated animal barriers; within-human barriers	Global	Public Health	Modelling	Cost per dog vaccinated; cost of PEP; cost per human life saved
Bechir (90)	2005	Chad	Multiple	Veterinary Health	2000	Domestic animal vaccination; vaccination for human populations with occupational exposure (nomadic herders)	Within-domesticated animal barriers; within-human barriers	National	Public Health; Agriculture	Process	Vaccination coverage in livestock and human populations; resource savings compared to conducting veterinary and public health vaccination campaigns separately; cost per child fully vaccinated
Roth (91)	2003	Mongolia	Brucellosis	Livestock and agriculture	N/A	Livestock vaccination	Within-domesticated animal barriers	National	N/A	Modelling	Cost-effectiveness relative to existing coverage (+)
United Against Rabies Collaboration (92)	2019	Global	Rabies	Public Health; Animal Health	2015	Domestic animal vaccination; integrated bite case management	Within-domesticated animal barriers;	Global	Public Health; Animal Health; Food	Cross-sectional; document review	Country adoption and implementation; PEP

First author	Year	Country	Disease(s)	Intervention sector	Implementation date	Policy type	Spillover pathway	Policy level	Governance sector	Study design	Key variables or concepts (Policy impact: direction of impact on outcome (+/-/=))
							human exposure; within-human barriers		and Agriculture		administration; bite reporting; cost; numbers of individuals receiving information about rabies
Berry (93)	2018	Global	Multiple	Global Public Health	N/A	Establishment of a global pandemic fund	Multiple points	Global	N/A	Modelling	Human health expenditures (-); lost productivity and commerce (-); self-insurance and protection capital (e.g., hospitals, surveillance networks, lab facilities and equipment) (+); outbreak risk (-)
Campbell (94)	2022	Australia	Multiple (Salmonella, E. Coli, T. gondii)	Livestock and hunting	1996; 1997; 2007; 2008; 2010; 2020	Standards for hygienic production of wild game meat	Pathogen prevalence in domesticated animals	National	Agriculture, fisheries and forestry	Qualitative interviews and document analysis	Private and public stakeholders involved; relevant legislation and standards; audit processes; policy learning and improvement
Campbell (94)	2022	South Africa	Avian influenza	Livestock and agriculture	1984; 2006; 2012	Test and quarantine	Pathogen prevalence in domesticated animals	National	Agriculture	Qualitative interviews and document analysis	Private and public stakeholders involved; relevant legislation and standards; audit processes; policy learning and improvement
Campbell (94)	2022	France	Multiple (including Q Fever, Salmonella, Brucellosis)	Animal Health; Hunting	Not stated	Veterinary inspection of wild-caught carcasses	Human exposure	National	Animal Health; hunting	Qualitative interviews and document analysis	Private and public stakeholders involved; relevant legislation and standards; audit processes; policy learning and improvement

First author	Year	Country	Disease(s)	Intervention sector	Implementation date	Policy type	Spillover pathway	Policy level	Governance sector	Study design	Key variables or concepts (Policy impact: direction of impact on outcome (+/-/=))
Ministry of Agriculture, Livestock, Fisheries and Cooperatives; and Ministry of Health (95)	2021	Kenya	Multiple (including rabies, brucellosis, tuberculosis, salmonella)	Animal Health; Public Health	2012	One Health strategic plan	Multiple points	National	Agriculture and livesotck; Health	Not stated	Agenda-setting; preparedness and response capacity; guideline development; challenges

References for included evaluations

1. Li Y, Wang Y, Shen C, Huang J, Kang J, Huang B, et al. Closure of live bird markets leads to the spread of H7N9 influenza in China. PLoS One [Internet]. 2018 Dec 12 [cited 2021 Apr 6];13(12). Available from: https://www.ncbi.nlm.nih.gov/pmc/articles/PMC6291110/

2. Abbas B, Yousif MA, Nur HM. Animal health constraints to livestock exports from the Horn of Africa: -EN- -FR- Restrictions sanitaires imposées aux exportations de bétail à partir de la corne de l'Afrique -ES- Limitaciones zoosanitarias a las exportaciones de ganado desde el Cuerno de África. Rev Sci Tech OIE. 2014 Dec 1;33(3):711–21.

3. Brennan A, Cross PC, Portacci K, Scurlock BM, Edwards WH. Shifting brucellosis risk in livestock coincides with spreading seroprevalence in elk. PLOS ONE. 2017 Jun 13;12(6):e0178780.

4. Fournié G, Guitian FJ, Mangtani P, Ghani AC. Impact of the implementation of rest days in live bird markets on the dynamics of H5N1 highly pathogenic avian influenza. Journal of The Royal Society Interface. 2011 Aug 7;8(61):1079–89.

5. Kung NY, Guan Y, Perkins NR, Bissett L, Ellis T, Sims L, et al. The Impact of a Monthly Rest Day on Avian Influenza Virus Isolation Rates in Retail Live Poultry Markets in Hong Kong. Avian Diseases. 2003 Sep;47(s3):1037–41.

6. Kung N, McLaughlin A, Taylor M, Moloney B, Wright T, Field H. Hendra Virus and Horse Owners – Risk Perception and Management. PLOS ONE. 2013 Nov 15;8(11):e80897.

7. Pinsent A, Pepin KM, Zhu H, Guan Y, White MT, Riley S. The persistence of multiple strains of avian influenza in live bird markets. Proceedings of the Royal Society B: Biological Sciences. 2017 Dec 6;284(1868):20170715.

8. Wu T, Perrings C, Shang C, Collins JP, Daszak P, Kinzig A, et al. Protection of wetlands as a strategy for reducing the spread of avian influenza from migratory waterfowl. Ambio. 2020 Apr 1;49(4):939–49.

9. Yu H, Wu JT, Cowling BJ, Liao Q, Fang VJ, Zhou S, et al. Effect of closure of live poultry markets on poultry-to-person transmission of avian influenza A H7N9 virus: an ecological study. The Lancet. 2014 Feb 8;383(9916):541–8.

10. Walker PGT, Cauchemez S, Metras R, Dung DH, Pfeiffer D, Ghani AC. A Bayesian Approach to Quantifying the Effects of Mass Poultry Vaccination upon the Spatial and Temporal Dynamics of H5N1 in Northern Vietnam. PLoS Comput Biol. 2010 Feb;6(2):e1000683.

11. Basinski AJ, Nuismer SL, Remien CH. A little goes a long way: Weak vaccine transmission facilitates oral vaccination campaigns against zoonotic pathogens. PLOS Neglected Tropical Diseases. 2019 Mar 8;13(3):e0007251.

12. Liu Z, Fang CT. A modeling study of human infections with avian influenza A H7N9 virus in mainland China. Int J Infect Dis. 2015 Dec;41:73–8.

13. Roy S, McElwain TF, Wan Y. A Network Control Theory Approach to Modeling and Optimal Control of Zoonoses: Case Study of Brucellosis Transmission in Sub-Saharan Africa. Gürtler RE, editor. PLoS Negl Trop Dis. 2011 Oct 11;5(10):e1259.

14. Häsler B, Howe KS, Hauser R, Stärk KDC. A qualitative approach to measure the effectiveness of active avian influenza virus surveillance with respect to its cost: A case study from Switzerland. Preventive Veterinary Medicine. 2012;105(3):209–22.

15. Horigan V, Gale P, Adkin A, Brown I, Clark J, Kelly L. A qualitative risk assessment of cleansing and disinfection requirements after an avian influenza outbreak in commercial poultry. British Poultry Science. 2019 Nov 2;60(6):691–9.

16. Hassim A, Dekker EH, Byaruhanga C, Reardon T, Van Heerden H. A retrospective study of anthrax on the Ghaap Plateau, Northern Cape province of South Africa, with special reference to the 2007–2008 outbreaks. Onderstepoort j vet res [Internet]. 2017 Sep 28 [cited 2021 Jul 22];84(1). Available from: https://ojvr.org/index.php/ojvr/article/view/1414

17. Selhorst T, Müller T. An evaluation of the efficiency of rabies control strategies in fox (Vulpes vulpes) populations using a computer simulation program. Ecological Modelling. 1999;124(2–3):221–32.

18. Samaan G, Hendrawati F, Taylor T, Pitona T, Marmansari D, Rahman R, et al. Application of a healthy food markets guide to two Indonesian markets to reduce transmission of "avian flu". Bull World Health Organ. 2012 Apr 1;90(4):295–300.

19. De Lucca T, Alves Rodrigues RC, Castagna C, Presotto D, De Nadai DV, Fagre A, et al. Assessing the rabies control and surveillance systems in Brazil: An experience of measures toward bats after the halt of massive vaccination of dogs and cats in Campinas, Sao Paulo. Prev Vet Med. 2013 Aug 1;111(1–2):126–33.

20. Hegazy YM, Ridler AL, Guitian FJ. Assessment and simulation of the implementation of brucellosis control programme in an endemic area of the Middle East. Epidemiol Infect. 2009 Oct;137(10):1436–48.

21. Busani L, Pozza MD, Bonfanti L, Toson M, Marangon S. Evaluation of the Efficacy of Intervention Measures and Vaccination for the Control of LPAI Epidemics in Verona Province (Veneto, Italy). Avian Diseases. 2007 Mar;51(s1):463–6.

22. Kimani T, Schelling E, Bett B, Ngigi M, Randolph T, Fuhrimann S. Public Health Benefits from Livestock Rift Valley Fever Control: A Simulation of Two Epidemics in Kenya. EcoHealth. 2016 Dec;13(4):729–42.

23. Cardador L, Tella JL, Anadon JD, Abellan P, Carrete M. The European trade ban on wild birds reduced invasion risks. Conserv Lett. 2019 May;12(3):e12631.

24. Bonwitt J, Dawson M, Kandeh M, Ansumana R, Sahr F, Brown H, et al. Unintended consequences of the 'bushmeat ban' in West Africa during the 2013–2016 Ebola virus disease epidemic. Social Science & Medicine. 2018 Mar;200:166–73.

25. Yuan J, Lau EHY, Li K, Leung YHC, Yang Z, Xie C, et al. Effect of Live Poultry Market Closure on Avian Influenza A(H7N9) Virus Activity in Guangzhou, China, 2014. Emerg Infect Dis. 2015 Oct;21(10):1784–93.

26. Naletoski I, Kirandziski T, Mitrov D, Krstevski K, Dzadzovski I, Acevski S. Gaps in Brucellosis Eradication Campaign in Sheep and Goats in Republic of Macedonia: Lessons Learned. Croat Med J. 2010 Aug;51(4):351–6.

27. Vivancos R, Showell D, Keeble B, Goh S, Kroese M, Lipp A, et al. Vaccination of Poultry Workers: Delivery and Uptake of Seasonal Influenza Immunization. Zoonoses Public Health. 2011 Mar;58(2):126–30.

28. Brinkley C, Kingsley JS, Mench J. A Method for Guarding Animal Welfare and Public Health: Tracking the Rise of Backyard Poultry Ordinances. J Community Health. 2018 Aug;43(4):639–46.

29. Smith GC, Cheeseman CL. A mathematical model for the control of diseases in wildlife populations: culling, vaccination and fertility control. Ecological Modelling. 2002 Apr;150(1–2):45–53.

30. Todd Weaver J, Malladi S, Bonney PJ, Patyk KA, Bergeron JG, Middleton JL, et al. A Simulation-Based Evaluation of Premovement Active Surveillance Protocol Options for the Managed Movement of Turkeys to Slaughter During an Outbreak of Highly Pathogenic Avian Influenza in the United States. Avian Diseases. 2016 May;60(1s):132–45.

31. Lauterbach SE, Nelson SW, Martin AM, Spurck MM, Mathys DA, Mollenkopf DF, et al. Adoption of recommended hand hygiene practices to limit zoonotic disease transmission at agricultural fairs. Preventive Veterinary Medicine [Internet]. 2020;182. Available from: https://www.scopus.com/inward/record.uri?eid=2-s2.0-85088938136&doi=10.1016%2fj.prevetmed.2020.105116&partnerID=40&md5=2a4ce113994ec8fcb5c2ffc81f55c728

32. Tustin J, Laberge K, Michel P, Reiersen J, Dadadottir S, Briem H, et al. A National Epidemic of Campylobacteriosis in Iceland, Lessons Learned. Zoonoses Public Health. 2011 Sep;58(6):440–7.

33. Lewis N, Dorjee S, Dube C, VanLeeuwen J, Sanchez J. Assessment of Effectiveness of Control Strategies Against Simulated Outbreaks of Highly Pathogenic Avian Influenza in Ontario, Canada. Transbound Emerg Dis. 2017 Jun;64(3):938–50.

34. Swayne DE, Pavade G, Hamilton K, Vallat B, Miyagishima K. Assessment of national strategies for control of high-pathogenicity avian influenza and low-pathogenicity notifiable avian influenza in poultry, with emphasis on vaccines and vaccination. Rev Sci Tech Off Int Epizoot. 2011 Dec;30(3):839–70.

35. Turkson PK, Okike I. Assessment of practices, capacities and incentives of poultry chain actors in implementation of highly pathogenic avian influenza mitigation measures in Ghana. Vet Med Sci. 2016 Feb;2(1):23–35.

36. Guerrier G, Foster H, Metge O, Chouvin C, Tui M. Cultural contexts of swine-related infections in Polynesia. Clinical Microbiology and Infection. 2013 Jul;19(7):595–9.

37. Anderson A, Shwiff S, Gebhardt K, Ramírez AJ, Shwiff S, Kohler D, et al. Economic Evaluation of Vampire Bat (*Desmodus rotundus)* Rabies Prevention in Mexico. Transbound Emerg Dis. 2014 Apr;61(2):140–6.

38. Akunzule AN, Koney EBM, Tiongco M. Economic impact assessment of highly pathogenic avian influenza on the poultry industry in Ghana. Worlds Poult Sci J. 2009 Sep;65(3):517–27.

39. Andronico A, Courcoul A, Bronner A, Scoizec A, Lebouquin-Leneveu S, Guinat C, et al. Highly pathogenic avian influenza H5N8 in south-west France 2016–2017: A modeling study of control strategies. Epidemics. 2019 Sep;28:100340.

40. Wu P, Jiang H, Wu JT, Chen E, He J, Zhou H, et al. Poultry Market Closures and Human Infection with Influenza A(H7N9) Virus, China, 2013-14. Emerg Infect Dis. 2014 Nov;20(11):1891–4.

41. Kang M, He J, Song T, Rutherford S, Wu J, Lin J, et al. Environmental Sampling for Avian Influenza A(H7N9) in Live-Poultry Markets in Guangdong, China. Davis T, editor. PLoS ONE. 2015 May 1;10(5):e0126335.

42. Beyer HL, Hampson K, Lembo T, Cleaveland S, Kaare M, Haydon DT. Metapopulation dynamics of rabies and the efficacy of vaccination. Proc R Soc B-Biol Sci. 2011 Jul 22;278(1715):2182–90.

43. Chowell G, Simonsen L, Towers S, Miller MA, Viboud C. Transmission potential of influenza A/H7N9, February to May 2013, China. BMC Med. 2013 Oct 2;11:214.

44. Backer JA, van Roermund HJW, Fischer EAJ, van Asseldonk MAPM, Bergevoet RHM. Controlling highly pathogenic avian influenza outbreaks: An epidemiological and economic model analysis. Prev Vet Med. 2015 Sep 1;121(1–2):142–50.

45. Brooks-Moizer F, Roberton SI, Edmunds K, Bell D. Avian Influenza H5N1 and the Wild Bird Trade in Hanoi, Vietnam. Ecol Soc. 2009 Jun;14(1):28.

46. Backer JA, Hagenaars TJ, van Roermund HJW, de Jong MCM. Modelling the effectiveness and risks of vaccination strategies to control classical swine fever epidemics. J R Soc Interface. 2009 Oct 6;6(39):849–61.

47. El Masry I, Rijks J, Peyre M, Taylor N, Lubroth J, Jobre Y. Modelling influenza A H5N1 vaccination strategy scenarios in the household poultry sector in Egypt. Trop Anim Health Prod. 2014 Jan;46(1):57–63.

48. Knight-Jones TJD, Gibbens J, Wooldridge M, Staerk KDC. Assessment of Farm-Level Biosecurity Measures after an Outbreak of Avian Influenza in the United Kingdom. Transbound Emerg Dis. 2011 Feb;58(1):69–75.

49. Xing Y, Song L, Sun GQ, Jin Z, Zhang J. Assessing reappearance factors of H7N9 avian influenza in China. Appl Math Comput. 2017 Sep 15;309:192–204.

50. Lin X, Zhang D, Wang X, Huang Y, Du Z, Zou Y, et al. Attitudes of consumers and live-poultry workers to central slaughtering in controlling H7N9: a cross-sectional study. BMC Public Health. 2017 May 26;17:517.

51. De Serres G, Skowronski DM, Mimault P, Ouakki M, Maranda-Aubut R, Duval B. Bats in the Bedroom, Bats in the Belfry: Reanalysis of the Rationale for Rabies Postexposure Prophylaxis. Clin Infect Dis. 2009 Jun 1;48(11):1493–9.

52. Shwiff SA, Sterner RT, Hale R, Jay MT, Sun B, Slate D. Benefit Cost Scenarios of Potential Oral Rabies Vaccination for Skunks in California. Journal of Wildlife Diseases. 2009 Jan;45(1):227–33.

53. Huang Z, Wang J, Zuo A. Chinese farmers' willingness to accept compensation to practice safe disposal of HPAI infected chicken. Prev Vet Med. 2017 Apr 1;139:67–75.

54. Hunter C, Birden HH, Toribio JA, Booy R, Abdurrahman M, Ambarawati AIGAA, et al. Community preparedness for highly pathogenic Avian influenza on Bali and Lombok, Indonesia. Rural Remote Health. 2014 Sep;14(3):2772.

55. Teng Y, Bi D, Guo X, Hu D, Feng D, Tong Y. Contact reductions from live poultry market closures limit the epidemic of human infections with H7N9 influenza. J Infect. 2018 Mar;76(3):295–304.

56. Karki S, Lupiani B, Budke CM, Karki NPS, Rushton J, Ivanek R. Cost-benefit analysis of avian influenza control in Nepal. OIE Revue Scientifique et Technique. 2015;34(3):813–27.

57. Kangas S, Lyytikäinen T, Peltola J, Ranta J, Maijala R. Costs of two alternative Salmonella control policies in Finnish broiler production. Acta Veterinaria Scandinavica [Internet]. 2007;49(1). Available from: https://www.scopus.com/inward/record.uri?eid=2-s2.0-38949119786&doi=10.1186%2f1751-0147-49-35&partnerID=40&md5=1a5a5cb9be107d9c92f10af6743ed9b7

58. Zhu G, Kang M, Wei X, Tang T, Liu T, Xiao J, et al. Different intervention strategies toward live poultry markets against avian influenza A (H7N9) virus: Model-based assessment. Environmental Research [Internet]. 2020; Available from: https://www.scopus.com/inward/record.uri?eid=2-s2.0-85096862816&doi=10.1016%2fj.envres.2020.110465&partnerID=40&md5=477f42ba6d643042adfe7a99bb2e3414

59. Stewart RJ, Rossow J, Conover JT, Lobelo EE, Eckel S, Signs K, et al. Do animal exhibitors support and follow recommendations to prevent transmission of variant influenza at agricultural fairs? A survey of animal exhibitor households after a variant influenza virus outbreak in Michigan. Zoonoses Public Health. 2018 Feb;65(1):195–201.

60. Oladokun AT, Meseko CA, Ighodalo E, John B, Ekong PS. Effect of intervention on the control of Highly Pathogenic Avian Influenza in Nigeria. :8.

61. Karabozhilova I, Wieland B, Alonso S, Salonen L, Häsler B. Backyard chicken keeping in the Greater London Urban Area: welfare status, biosecurity and disease control issues. British Poultry Science. 2012 Aug;53(4):421–30.

62. Manyweathers J, Field H, Longnecker N, Agho K, Smith C, Taylor M. 'Why won't they just vaccinate?' Horse owner risk perception and uptake of the Hendra virus vaccine. BMC Vet Res. 2017 Apr 13;13:103.

63. Sanchez JN, Hudgens BR. Vaccination and monitoring strategies for epidemic prevention and detection in the Channel Island fox (Urocyon littoralis). Seirin-Lee S, editor. PLoS ONE. 2020 May 18;15(5):e0232705.

64. Yee KS, Carpenter TE, Mize S, Cardona CJ. The Live Bird Market System and Low-Pathogenic Avian Influenza Prevention in Southern California. :5.

65. Huot C, De Serres G, Duval B, Maranda-Aubut R, Ouakki M, Skowronski DM. The cost of preventing rabies at any cost: Post-exposure prophylaxis for occult bat contact. Vaccine. 2008;26(35):4446–50.

66. Manyweathers J, Field H, Jordan D, Longnecker N, Agho K, Smith C, et al. Risk Mitigation of Emerging Zoonoses: Hendra Virus and Non-Vaccinating Horse Owners. Transbound Emerg Dis. 2017 Dec;64(6):1898–911.

67. Wang W, Artois J, Wang X, Kucharski AJ, Pei Y, Tong X, et al. Effectiveness of live poultry market interventions on human infection with avian influenza A(H7N9) virus, China. Emerging Infectious Diseases. 2020;26(5):891–901.

68. Mroz C, Gwida M, El-Ashker M, Ziegler U, Homeier-Bachmann T, Eiden M, et al. Rift Valley fever virus infections in Egyptian cattle and their prevention. Transbound Emerg Dis. 2017 Dec;64(6):2049–58.

69. Davis AJ, Nelson KM, Kirby JD, Wallace R, Ma X, Pepin KM, et al. Rabies surveillance identifies potential risk corridors and enables management evaluation. Viruses [Internet]. 2019;11(11). Available from: https://www.scopus.com/inward/record.uri?eid=2-s2.0-85074547341&doi=10.3390%2fv11111006&partnerID=40&md5=7e257e5e1e407b60ed2127b3b5d92e50

70. Kwan NCL, Ogawa H, Yamada A, Sugiura K. Quantitative risk assessment of the introduction of rabies into Japan through the illegal landing of dogs from Russian fishing boats in the ports of Hokkaido, Japan. Prev Vet Med. 2016 Jun 1;128:112–23.

71. García-Díaz P, Ross JV, Woolnough AP, Cassey P. Managing the risk of wildlife disease introduction: pathway-level biosecurity for preventing the introduction of alien ranaviruses. Bellard C, editor. J Appl Ecol. 2017 Feb;54(1):234–41.

72. Graiver DA, Topliff CL, Kelling CL, Bartelt-Hunt SL. Survival of the avian influenza virus (H6N2) after land disposal. Environmental Science and Technology. 2009;43(11):4063–7.

73. Chen Y, Cheng J, Xu Z, Hu W, Lu J. Live poultry market closure and avian influenza A (H7N9) infection in cities of China, 2013-2017: An ecological study. BMC Infectious Diseases [Internet]. 2020;20(1). Available from: https://www.scopus.com/inward/record.uri?eid=2-s2.0-85085389244&doi=10.1186%2fs12879-020-05091-7&partnerID=40&md5=3d0b44da21fd54ab040ad844efe19a93

74. Fournie G, Guitian J, Desvaux S, Cuong VC, Dung DH, Pfeiffer DU, et al. Interventions for avian influenza A (H5N1) risk management in live bird market networks. Proceedings of the National Academy of Sciences of the United States of America. 2013;110(22):9177–82.

75. Ma J, Yang N, Gu H, Bai L, Sun J, Gu S, et al. Effect of closure of live poultry markets in China on prevention and control of human infection with H7N9 avian influenza: a case study of four cities in Jiangsu Province. Journal of Public Health Policy. 2019;40(4):436–47.

76. Thomas D, Delgado A, Louison B, Lefrancois T, Shaw J. Examining dog owners' beliefs regarding rabies vaccination during government-funded vaccine clinics in Grenada to improve vaccine coverage rates. Preventive Veterinary Medicine. 2013;110(3–4):563–9.

77. Gordon ER, Krebs JW, Rupprecht CR, Real LA, Childs JE. Persistence of elevated rabies prevention costs following post-epizootic declines in rates of rabies among raccoons (Procyon lotor). Preventive Veterinary Medicine. 2005;68(2–4):195–222.

78. Mendez D, Buttner P, Speare R. Hendra virus in Queensland, Australia, during the winter of 2011: Veterinarians on the path to better management strategies. Prev Vet Med. 2014 Nov 1;117(1):40–51.

79. Okello AL, Thomas LF. Human taeniasis: current insights into prevention and management strategies in endemic countries. RISK MANAG HEALTHC POLICY. 2017;10:107–16.

80. Lu H, Ismail MM, Khan OA, Al Hammad Y, Rhman SSA, Al-Blowi MH. Epidemic Outbreaks, Diagnostics, and Control Measures of the H5N1 Highly Pathogenic Avian Influenza in the Kingdom of Saudi Arabia, 2007-08. Avian Dis. 2010 Mar;54(1):350–6.

81. Rasouli J, Holakoui K, Forouzanfar MH, Salari S, Bahoner, Rashidian A. Cost effectiveness of livestock vaccination for brucellosis in West-Azerbayjan province. Urmia Medical Journal. 2009;20(1):Pe13-En77.

82. Wilson PJ, Clark KA. Postexposure rabies prophylaxis protocol for domestic animals and epidemiologic characteristics of rabies vaccination failures in Texas: 1995-1999. Journal of the American Veterinary Medical Association. 2001 Feb;218(4):522–5.

83. Wang Man, Mao YunXia, Luo Le, Chen XueQin, Li Lei. Effects of short-term closing live poultry markets in controlling the H7N9 avian influenza pollution. Chinese Journal of Zoonoses. 2018;34(1):79–84.

84. Lyu HuaKun, Gong ZhenYu, Sun JiMin, Kong LingJie, Chen ZhiPing. Epidemic characteristics of human infection with avian influenza A (H7N9) virus and influence of closure of live poultry markets on the epidemic in Zhejiang. Disease Surveillance. 2014;29(9):700–3.

85. Cuthbert R, Taggart MA, Prakash V, Saini M, Swarup D, Upreti S, et al. Effectiveness of Action in India to Reduce Exposure of Gyps Vultures to the Toxic Veterinary Drug Diclofenac. Willis SG, editor. PLoS ONE. 2011 May 11;6(5):e19069.

86. John Weaver, Julie Punderson, John Stratton. OIE PVS Evaluation report of the Veterinary Services of Japan [Internet]. World Organization for Animal Health; 2016 [cited 2022 Sep 21]. Available from: https://www.woah.org/fileadmin/Home/eng/Support_to_OIE_Members/docs/pdf/20180727_Final_OIE_JAPAN_PVS_REPORT.pdf

87. Amparo ACB, Jayme SI, Roces MCR, Quizon MCL, Mercado MLL, Dela Cruz MPZ, et al. The evaluation of Animal Bite Treatment Centers in the Philippines from a patient perspective. Rupprecht CE, editor. PLoS ONE. 2018 Jul 26;13(7):e0200873.

88. Ferguson EA, Hampson K, Cleaveland S, Consunji R, Deray R, Friar J, et al. Heterogeneity in the spread and control of infectious disease: consequences for the elimination of canine rabies. Sci Rep. 2015 Dec;5(1):18232.

89. Shwiff SA, Hatch B, Anderson A, Nel LH, Leroux K, Stewart D, et al. Towards Canine Rabies Elimination in KwaZulu–Natal, South Africa: Assessment of Health Economic Data. Transboundary and Emerging Diseases. 2016;63(4):408–15.

90. Bechir M, Schelling E, Wyss K, Daugla DM, Daoud S, Tanner M, et al. APPROCHE NOVATRICE DES VACCINATIONS EN SANTÉ PUBLIQUE ET EN MÉDECINE VÉTÉRINAIRE CHEZ LES PASTEURS NOMADES AU TCHAD: EXPÉRIENCES ET COÛTS [An innovative approach

combining human and animal vaccination campaigns in nomadic settings of Chad: experiences and costs]. Médecine Tropicale. 2005;64(5):6.

91. Roth F, Zinsstag J, Orkhon D, Chimed-Ochir G, Hutton G, Cosivi O, et al. Human health benefits from livestock vaccination for brucellosis: case study. Bulletin of the World Health Organization. 2003;17.

92. World Health Organization, Food and Agriculture Organization of the United Nations, World Organisation for Animal Health. Zero by 30: the global strategic plan to end human deaths from dog-mediated rabies by 2030 [Internet]. Geneva: World Health Organization; 2018 [cited 2022 Sep 21]. 47 p. Available from: https://apps.who.int/iris/handle/10665/272756

93. Berry K, Allen T, Horan RD, Shogren JF, Finnoff D, Daszak P. The Economic Case for a Pandemic Fund. EcoHealth. 2018 Jun;15(2):244–58.

94. Sam Campbell, Anastasiya Timoshyna, Glenn Sant, Duan Biggs, Alexander Braczkowski, Hernan Caceres-Escobar, et al. REVIEW: OPTIONS FOR MANAGING AND TRACING WILD ANIMAL TRADE CHAINS TO REDUCE ZOONOTIC DISEASE RISK [Internet]. Cambridge, UK: TRAFFIC UK; 2022 [cited 2022 Sep 23]. Available from: https://www.traffic.org/site/assets/files/18246/traps_2_supply_chain_management_traceability_review_report_2-1.pdf

95. Ministry of Agriculture, Livestock, Fisheries and Cooperatives, Ministry of Health. Zoonotic Disease Unit: One Health Strategic Plan for the Prevention and Control of Zoonotic Diseases in Kenya (2021-2025) [Internet]. Nairobi; 2021 [cited 2022 Sep 27]. Available from: https://www.onehealthcommission.org/documents/filelibrary/resources/one_health_strategic_action_plans/OneHealthStrategicPlan_Kenya_202120_8756689A2C54E.pdf
