# Supplementary material for: Policies to prevent zoonotic spillover: a systematic scoping review of evaluative evidence
Source: Global Health. 2023 Nov 8;19:82. doi: 10.1186/s12992-023-00986-x (PMC10634115; doi:10.1186/s12992-023-00986-x)
Supplement: Supplementary file 4 — Supplementary Material 4 [file 12992_2023_986_MOESM4_ESM.pdf]

Supplementary file 2

Data charting form

**Record**	Author(s)
	Year
**Policy**	Country
	World region (World Bank grouping) (126)
	Country income (World Bank grouping) (126)
	Disease
	Non-government stakeholder or sector responsible for implementing policy (retail, agriculture, conservation, etc.)
	Implementation date (start date, or range if the policy has been changed)
	Intervention type
	Location along spillover pathway adapted from Plowright et al. (8,22)
	Policy level (local, national, regional, global)
	Multi-sectoral initiative (Y/N)
	Government sector(s) responsible for policy
**Evaluation**	Aim
	Evaluation type (formative, process, outcome, economic, impact (127))
	Study design (for natural experiments, categorise using typology developed by Leatherdale (128))
	Theoretical framework and/or logic model underpinning evaluation (if described)
	Period of observation
	Variables of interest: Outcome measure(s) and change in measure(s) (+/-/=) for quantitative impact evaluations; measures or observed variables or concepts for other types of study
	Consideration of unintended consequences (Y/N)
	If yes, which unintended consequences? (e.g., economic outcomes, food security)
