# Supplementary material for: Policies to prevent zoonotic spillover: a systematic scoping review of evaluative evidence
Source: Global Health. 2023 Nov 8;19:82. doi: 10.1186/s12992-023-00986-x (PMC10634115; doi:10.1186/s12992-023-00986-x)
Supplement: Supplementary file 5 — Supplementary Material 5 [file 12992_2023_986_MOESM5_ESM.pdf]

Supplementary file 1

Academic database search

<u>Example search strategy highlighting key concepts</u>

Scopus search, all terms in TITLE-ABS-KEY; Block 1 AND 2 AND 3 AND 4

Block 1 – Policy interventions

policy OR law OR legal OR legislat* OR regulat* OR tariff OR subsidy OR tax OR ban OR "voluntary agreement" OR incentive OR fiscal OR guidelines OR govern* OR federal* OR closure OR closing OR state* OR "rest day*" OR "border control*" OR "habitat protection" OR "wetland protection" OR "supplement* fed" OR "supplement* feed*" OR "market size"

Block 2 – Prevention

Prevent* OR "ecological intervention*" OR "non-pharmaceutical intervention*" OR "public health" OR "risk management" OR "risk minimisation" OR "control strateg*" OR "outbreak risk" OR "reduc* W/5 transmission" OR "reduc* W/5 infection"

Block 3 - Zoonoses

Zika OR ebola OR covid-19 OR sars-cov-2 OR coronavirus OR sars OR mers OR h1n1 OR h7n9 OR h5n1 OR "one health" OR dengue OR "nipah virus" OR influenza OR zoonoses OR zoonosis OR zoonotic OR "West Nile" OR "HIV/AIDS" OR "avian flu" OR "hendra virus" OR "marburg virus" OR "yellow fever" OR "tick-borne encephalitis" OR "emerging infectious diseases" OR "emergent infectious diseases" OR brucellosis OR rabies OR chikungunya OR "bovine spongiform encephalopathy" OR "rift valley fever"

Block 4 – Spillover events

Spillover OR "spill over" OR "cross-species transmission" OR poultry OR wildlife OR bushmeat OR "bush meat" OR livestock OR "animal market*" OR "wet market*" OR "bird market*" OR horse* OR waterfowl OR fowl OR bat OR bats OR mammal* OR swine OR pig* OR poaching OR "pet trade" OR pork OR "trade W/5 animal"

<u>Search strings for all included academic databases</u>

Scopus search

TITLE-ABS-KEY(Zika OR ebola OR covid-19 OR sars-cov-2 OR coronavirus OR sars OR mers OR h1n1 OR h7n9 OR h5n1 OR "one health" OR dengue OR "nipah virus" OR influenza OR

zoonoses OR zoonosis OR zoonotic OR “West Nile” OR “HIV/AIDS” OR “avian flu” OR “hendra virus” OR “marburg virus” OR “yellow fever” OR “tick-borne encephalitis” OR “emerging infectious diseases” OR “emergent infectious diseases” OR brucellosis OR rabies OR chikungunya OR “bovine spongiform encephalopathy” OR “rift valley fever”) AND TITLE-ABS-KEY(Spillover OR “spill over” OR “cross-species transmission” OR poultry OR wildlife OR bushmeat OR “bush meat” OR livestock OR “animal market*” OR “wet market*” OR “bird market*” OR horse* OR waterfowl OR fowl OR bat OR bats OR mammal* OR swine OR pig* OR poaching OR “pet trade” OR pork OR “trade W/5 animal”) AND TITLE-ABS-KEY(policy OR law OR legal OR legislat* OR regulat* OR tariff OR subsidy OR tax OR ban OR “voluntary agreement” OR incentive OR fiscal OR guidelines OR govern* OR federal* OR closure OR closing OR state* OR “rest day*” OR “border control*” OR “habitat protection” OR “wetland protection” OR “supplement* fed” OR “supplement* feed*” OR “market size”) AND TITLE-ABS-KEY(Prevent* OR “ecological intervention*” OR “non-pharmaceutical intervention*” OR “public health” OR “risk management” OR “risk minimisation” OR “control strateg*” OR “outbreak risk” OR “reduc* W/5 transmission” OR “reduc* W/5 infection”)

Medline search

(((Zika[Title/Abstract] OR ebola[Title/Abstract] OR covid-19[Title/Abstract] OR sars-cov-2[Title/Abstract] OR coronavirus[Title/Abstract] OR sars[Title/Abstract] OR mers[Title/Abstract] OR h1n1[Title/Abstract] OR h7n9[Title/Abstract] OR h5n1[Title/Abstract] OR "one health"[Title/Abstract] OR dengue[Title/Abstract] OR "nipah virus"[Title/Abstract] OR influenza[Title/Abstract] OR zoonoses[Title/Abstract] OR zoonosis[Title/Abstract] OR zoonotic[Title/Abstract] OR "West Nile"[Title/Abstract] OR "HIV/AIDS"[Title/Abstract] OR "avian flu"[Title/Abstract] OR "hendra virus"[Title/Abstract] OR "marburg virus"[Title/Abstract] OR "yellow fever"[Title/Abstract] OR "tick-borne encephalitis"[Title/Abstract] OR "emerging infectious diseases"[Title/Abstract] OR "emergent infectious diseases"[Title/Abstract] OR brucellosis[Title/Abstract] OR rabies[Title/Abstract] OR chikungunya[Title/Abstract] OR "bovine spongiform encephalopathy"[Title/Abstract] OR "rift valley fever"[Title/Abstract] OR zoonoses [mesh]) AND (Spillover[Title/Abstract] OR "spill over"[Title/Abstract] OR "cross-species transmission"[Title/Abstract] OR poultry[Title/Abstract] OR wildlife[Title/Abstract] OR bushmeat[Title/Abstract] OR "bush meat"[Title/Abstract] OR livestock[Title/Abstract] OR "animal market"[Title/Abstract] OR "animal markets"[Title/Abstract] OR "wet market"[Title/Abstract] OR "wet markets"[Title/Abstract] OR "bird market"[Title/Abstract] OR "bird markets"[Title/Abstract] OR horse[Title/Abstract] OR horses[Title/Abstract] OR waterfowl[Title/Abstract] OR

fowl[Title/Abstract] OR bat[Title/Abstract] OR bats[Title/Abstract] OR mammal[Title/Abstract] OR mammals[Title/Abstract] OR mammalian[Title/Abstract] OR swine[Title/Abstract] OR pig[Title/Abstract] OR pigs[Title/Abstract] OR poaching[Title/Abstract] OR "pet trade"[Title/Abstract] OR pork[Title/Abstract] OR animal N5 trade[Title/Abstract] OR disease reservoir [mesh])) AND (policy[Title/Abstract] OR law[Title/Abstract] OR legal[Title/Abstract] OR legislation[Title/Abstract] OR legislative[Title/Abstract] OR legislating[Title/Abstract] OR regulation[Title/Abstract] OR regulations[Title/Abstract] OR regulatory[Title/Abstract] OR tariff[Title/Abstract] OR subsidy[Title/Abstract] OR tax[Title/Abstract] OR ban[Title/Abstract] OR "voluntary agreement"[Title/Abstract] OR incentive[Title/Abstract] OR fiscal[Title/Abstract] OR guidelines[Title/Abstract] OR government[Title/Abstract] OR governments[Title/Abstract] OR federal[Title/Abstract] OR federally[Title/Abstract] OR closure[Title/Abstract] OR closing[Title/Abstract] OR state[Title/Abstract] OR "rest day"[Title/Abstract] OR "rest days"[Title/Abstract] OR "border control"[Title/Abstract] OR "border controls"[Title/Abstract] OR "habitat protection"[Title/Abstract] OR "wetland protection"[Title/Abstract] OR "supplemental feeding"[Title/Abstract] OR "market size"[Title/Abstract])) AND (Prevent[Title/Abstract] OR prevention[Title/Abstract] OR "ecological intervention"[Title/Abstract] OR "ecological interventions"[Title/Abstract] OR "non-pharmaceutical intervention"[Title/Abstract] OR "non-pharmaceutical interventions"[Title/Abstract] OR "public health"[Title/Abstract] OR "risk management"[Title/Abstract] OR "risk minimisation"[Title/Abstract] OR "control strategy"[Title/Abstract] OR "control strategies"[Title/Abstract] OR "outbreak risk"[Title/Abstract] OR reducing N5 transmission[Title/Abstract] OR reducing N5 infection[Title/Abstract])

Web of knowledge search

AB=(Zika OR ebola OR covid-19 OR sars-cov-2 OR coronavirus OR sars OR mers OR h1n1 OR h7n9 OR h5n1 OR “one health” OR dengue OR “nipah virus” OR influenza OR zoonoses OR zoonosis OR zoonotic OR “West Nile” OR “HIV/AIDS” OR “avian flu” OR “hendra virus” OR “marburg virus” OR “yellow fever” OR “tick-borne encephalitis” OR “emerging infectious diseases” OR “emergent infectious diseases” OR brucellosis OR rabies OR chikungunya OR “bovine spongiform encephalopathy” OR “rift valley fever”)

AND

AB=(Spillover OR “spill over” OR “cross-species transmission” OR poultry OR wildlife OR bushmeat OR “bush meat” OR livestock OR “animal market*” OR “wet market*” OR “bird market*” OR horse* OR waterfowl OR fowl OR bat OR bats OR mammal* OR swine OR pig* OR poaching OR “pet trade” OR pork OR trade NEAR animal)

AND

AB=(policy OR law OR legal OR legislat* OR regulat* OR tariff OR subsidy OR tax OR ban OR “voluntary agreement” OR incentive OR fiscal OR guidelines OR govern* OR federal* OR closure OR closing OR state* OR “rest day*” OR “border control*” OR “habitat protection” OR “wetland protection” OR “supplement* fed” OR “supplement* feed*” OR “market size”)

AND

AB=(Prevent* OR “ecological intervention*” OR “non-pharmaceutical intervention*” OR “public health” OR “risk management” OR “risk minimisation” OR “control strateg*” OR “outbreak risk” OR reduc* NEAR transmission OR reduc* NEAR infection)

Ovid Global Health database search (all in abstract)

Zika OR ebola OR covid-19 OR sars-cov-2 OR coronavirus OR sars OR mers OR h1n1 OR h7n9 OR h5n1 OR one health OR dengue OR nipah virus OR influenza OR zoonoses OR zoonosis OR zoonotic OR West Nile OR HIV/AIDS OR avian flu OR hendra virus OR marburg virus OR yellow fever OR tick-borne encephalitis OR emerging infectious diseases OR emergent infectious diseases OR brucellosis OR rabies OR chikungunya OR bovine spongiform encephalopathy OR rift valley fever

AND

Spillover OR spill over OR cross-species transmission OR poultry OR wildlife OR bushmeat OR bush meat OR livestock OR animal market* OR wet market* OR bird market* OR horse* OR waterfowl OR fowl OR bat OR bats OR mammal* OR swine OR pig* OR poaching OR pet trade OR pork OR (trade adj5 animal)

AND

policy OR law OR legal OR legislat* OR regulat* OR tariff OR subsidy OR tax OR ban OR voluntary agreement OR incentive OR fiscal OR guidelines OR govern* OR federal* OR closure OR closing OR state* OR rest day* OR border control* OR habitat protection OR wetland protection OR supplement* fed OR supplement* feed* OR market size

AND

Prevent* OR ecological intervention* OR non-pharmaceutical intervention* OR public health OR risk management OR risk minimisation OR control strateg* OR outbreak risk OR (reduc* adj5 transmission) OR (reduc* adj5 infection)

Grey literature search

We searched the websites of 18 relevant organizations for grey literature reports on September 27, 2022. For each website, the following search was used in the search engine Google in an 'incognito' window:

evaluation policy "zoonotic disease" OR "emerging infectious disease" OR "spillover" OR "zoonoses" site:[organization domain name] filetype:pdf

The first 30 search results for each website were screened for inclusion. This number was chosen after piloting the search and identifying that the relevance of search results was relatively low after the first 30. Where fewer than 30 results were returned, all search results were screened. Where relevant evaluations were mentioned (e.g., as a case study within a broader report), reference lists were hand searched for more detailed documents describing the evaluation.

The websites of the following organizations were searched (numbers in brackets indicate results screened; results included):

1. World Organization for Animal Health (formerly OIE) 30; 2
2. Food and Agriculture Organization 30; 0
3. World Health Organization 30; 0
4. Wildlife Disease Association 30; 0
5. International Alliance against Health Risks in Wildlife Trade 2; 0
6. United Nations Environment Program 0; 0
7. United Nations Office for Drugs and Crime 30; 0
8. Global Alliance for Rabies Control 30; 6
9. EcoHealth Alliance 30; 1
10. Network for EcoHealth and One Health 0;0
11. International Livestock Research Institute 0;0
12. Preventing Pandemics at the Source 0;0
13. World Veterinary Association 25;0
14. CITES 22; 0
15. TRAFFIC 21;1
16. One Health Commission 30;1
17. World Wildlife Fund 0;0

18. World Trade Organization 30; 0 (N.B. The term ‘spillover’ was omitted from the search of the World Trade Organization’s website, as the term’s frequent use in economic or monetary contexts meant many irrelevant results were returned.)
